# Supplementary material for: Engineering selective competitors for the discrimination of highly conserved protein-protein interaction modules
Source: Nat Commun. 2019 Oct 4;10:4521. doi: 10.1038/s41467-019-12528-4 (PMC6778148; doi:10.1038/s41467-019-12528-4)
Supplement: Supplementary file 1 — Supplementary Information [file 41467_2019_12528_MOESM1_ESM.pdf]

Supplementary Information

Engineering selective competitors for the discrimination of highly conserved protein-protein interaction modules

Charlotte Rimbault[1,2], Kashyap Maruthi[3,4], Christelle Breillat[1,2], Camille Genuer[1,2], Sara Crespillo[1,2], Virginia Puente-Muñoz[1,2], Ingrid Chamma[1,2], Isabel Gauthereau[1,2], Ségolene Antoine[1,2], Coraline Thibaut[1,2,] Fabienne Wong Jun Tai[5], Benjamin Dartigues[5], Dolors Grillo-Bosch[1,2], Stéphane Claverol[6], Christel Poujol[7], Daniel Choquet[1,2,7], Cameron D. Mackereth[3,4*] & Matthieu Sainlos[1,2*].

[1]Interdisciplinary Institute for Neuroscience, UMR 5297, Centre National de la Recherche Scientifique, F-33076 Bordeaux, France
[2]Interdisciplinary Institute for Neuroscience, University of Bordeaux, F-33076 Bordeaux, France
[3]Univ. Bordeaux, Institut Européen de Chimie et Biologie, 2 rue Robert Escarpit, F-33607 Pessac, France
[4]Inserm U1212, CNRS UMR 5320, ARNA Laboratory, Univ. Bordeaux, 146 rue Léo Saignat, F-33076 Bordeaux, France
[5]University of Bordeaux, CBiB-LaBRI, F-33000 Bordeaux, France
[6]Proteome Platform, Functional Genomic Center of Bordeaux, University of Bordeaux, F-33076 Bordeaux, France
[7]Bordeaux Imaging Center, UMS 3420 Centre National de la Recherche Scientifique, University of Bordeaux, US 4 INSERM, F-33076 Bordeaux, France

Correspondence and requests for materials should be addressed to
M.S. (sainlos@u-bordeaux.fr)

a

b

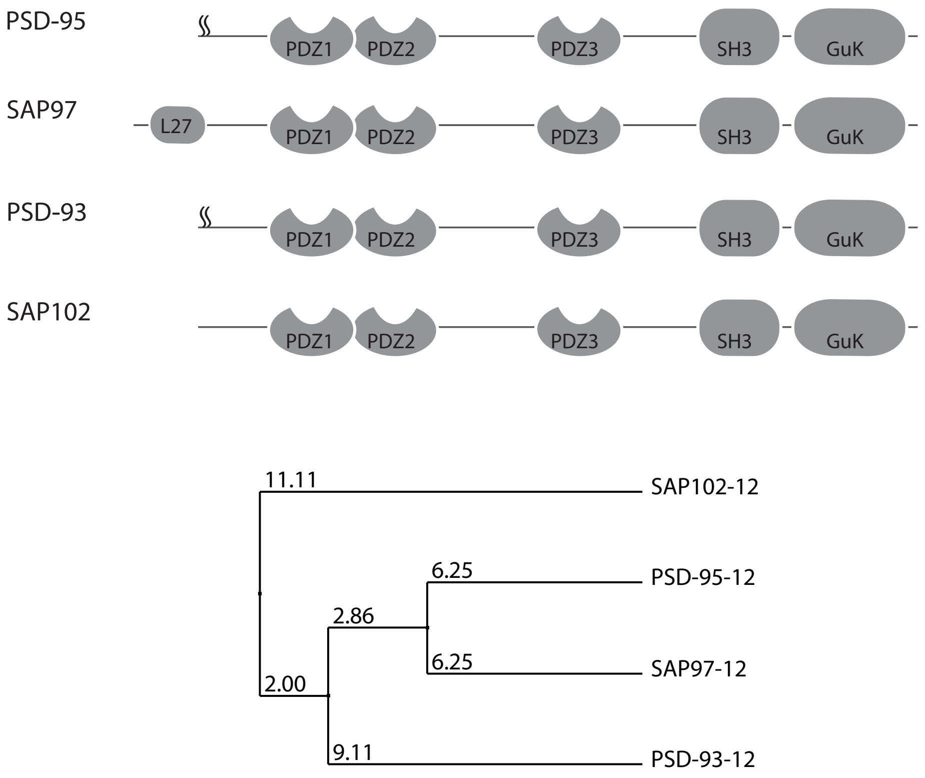

Supplementary Figure 1 | (a) Domain organization of PSD-95 family members. PSD-95 and PSD-93 can both be doubly palmitoylated on their N-terminus. PDZ: PSD-95, Discs large, Zona occludens 1 domain; SH3: SRC Homology 3 domain; GuK: Guanylate Kinase-like domain; L27: Lin2, Lin7 domain. **(b)** Average distance of the tandem PDZ domains sequence using percentage identity (obtained with Jalview).

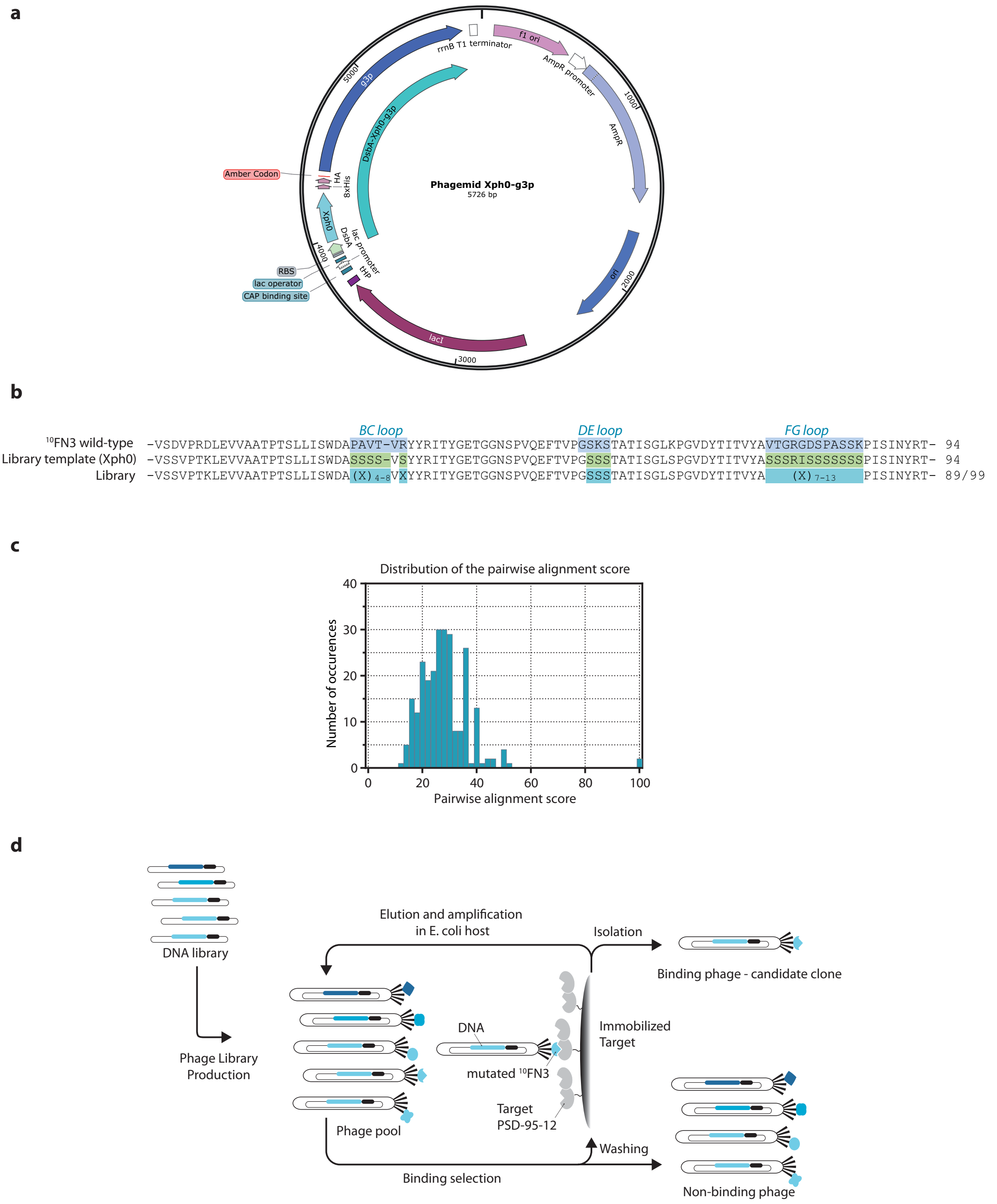

Supplementary Figure 2 | (a) Map and features of the modified phagemid used in this study (derived from pSEX81). **(b)** Sequence alignment of [10]FN3 wild-type, the template used for creating the library (referred to as Xph0) and the general design of the library used in this study. X represents any amino acid as well as the amber stop codon (NNK degenerate codons used for diversification). **(c)** Evaluation of the library quality by analyzing the score distribution of the pairwise alignment of 96 randomly picked colonies sequences (obtained with SynDivA). The general low scores indicate -by projection over 96 colonies- high diversity of the sequences and minimal number of identical within the library. Mean of the pairwise alignment 27.81 ± 10.12 (s.d.). **(d)** General principle of the phage display selection used against PSD-95 tandem PDZ domains 1 and 2.

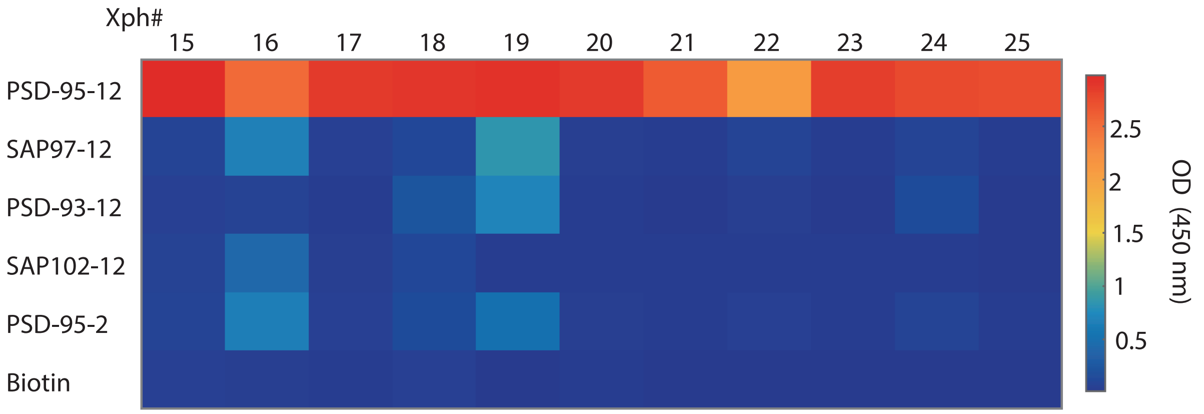

Supplementary Figure 3 | Phage-ELISA of Xph15-25 against biotinylated targets. Each value represents the average of two independent experiments in which two different colonies were used for each Xph clone. Absence of response for the wells in which PDZ domains are substituted by biotin indicate that the clones are specifically recognizing PDZ domains and not other elements required for the selection (streptavidin) or the Phage-ELISA (plastic). Source data are provided as Source Data file.

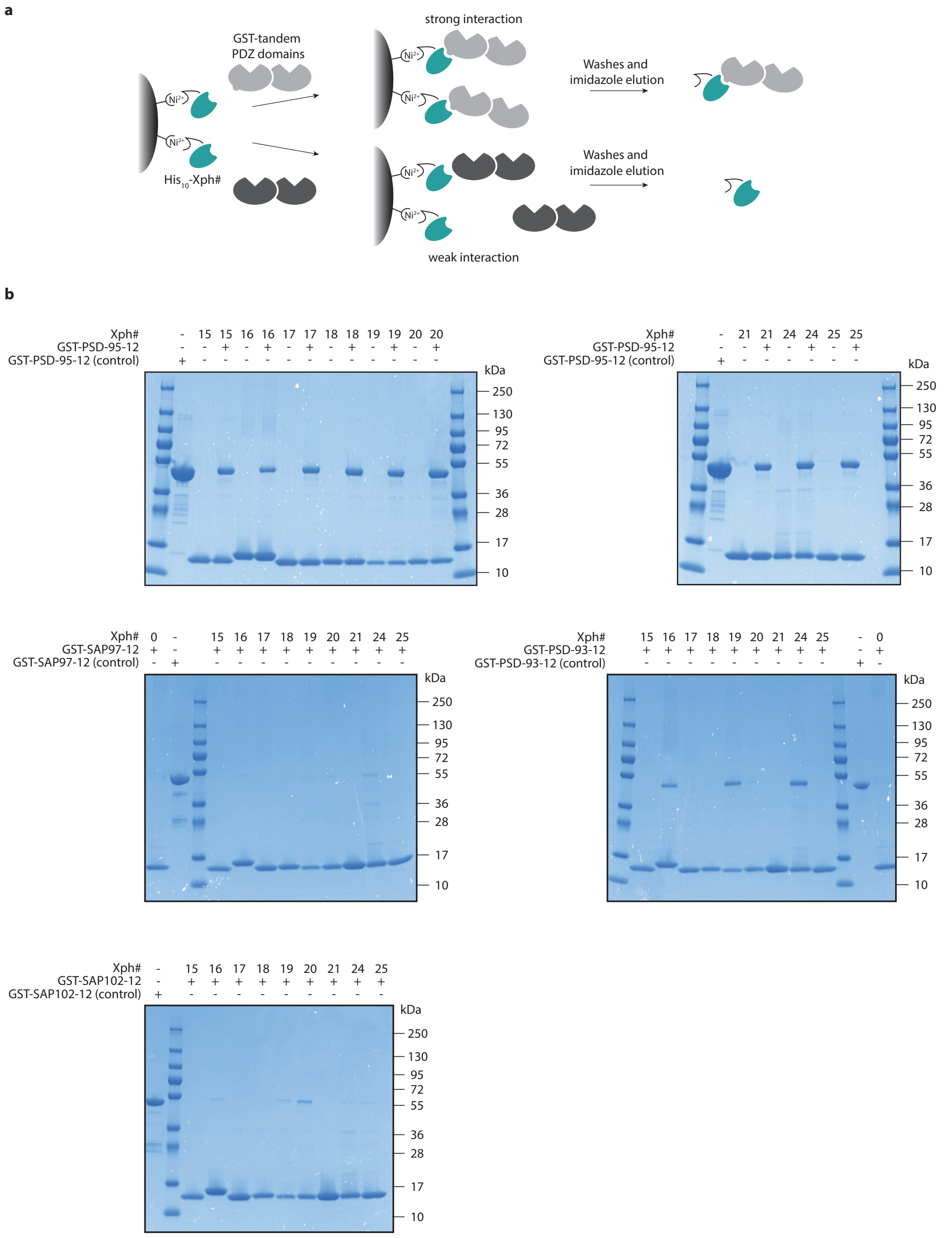

Supplementary Figure 4 | (a) Principle of the pull-down. Xph constructs were directly isolated from E coli lysates with Ni-NTA magnetic beads. The beads functionalized with Xph were next incubated with purified recombinant GST fusion of the tandem PDZ domains and washed. Proteins left on the beads after the washes were eluted with 500 mM imidazole and analyzed by SDS-PAGE. **(b)** Colloidal blue-stained SDS-PAGE analysis of eluted material. Xph clones are running between 10-17 kDa, GST-fusions of the tandem PDZ domains are running between 50-55 kDa. All studied Xph clones were obtained with comparable yields. Densitometric analysis of the pull-down efficiencies is performed by calculating the ratio from the band intensities of the bound tandem PDZ domains and the immobilized Xph. Experiments were performed in duplicate (PSD-95-12) or once (SAP97, SAP102 and PSD-93).

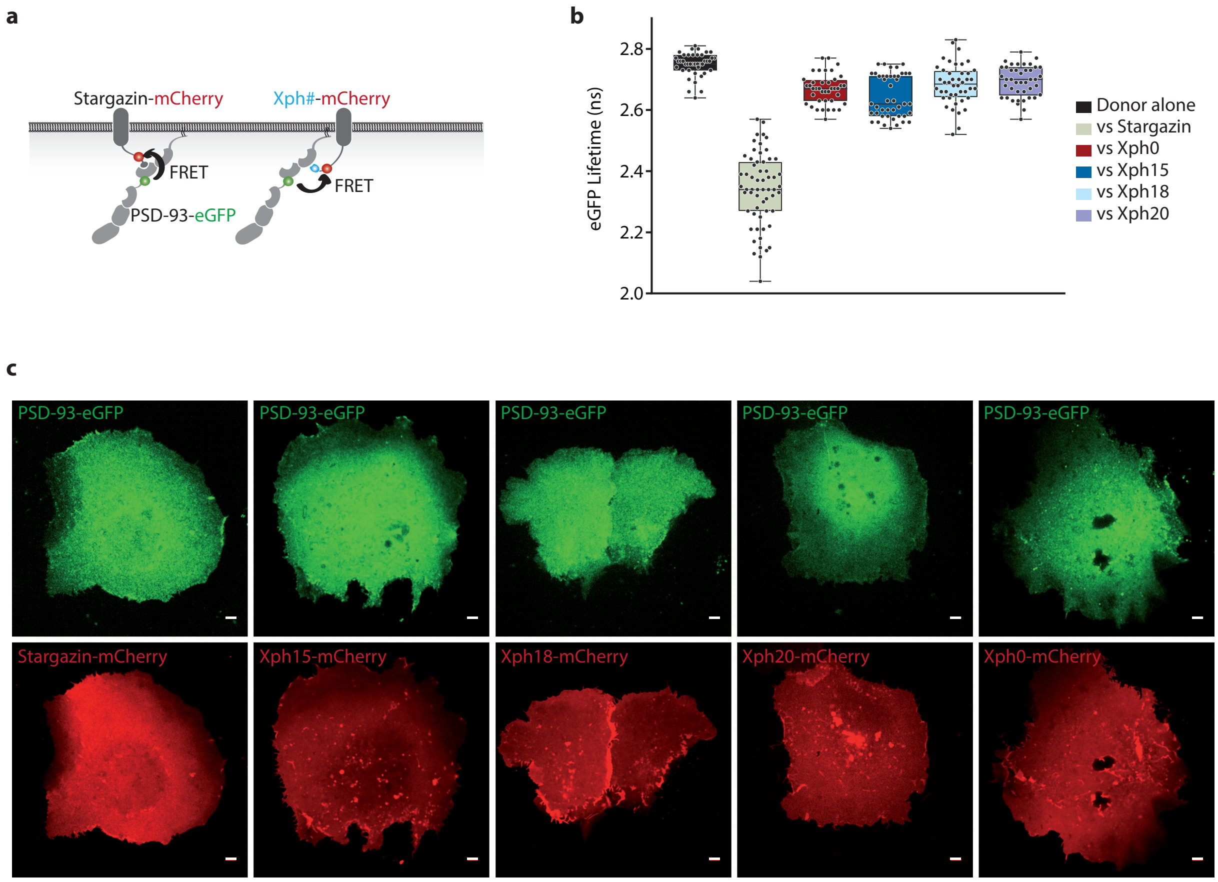

Supplementary Figure 5 | (a) Schematic of the FRET systems used for measurement of the donor lifetime (FLIM). **(b)** Lifetime of eGFP inserted in PSD-93 in presence of the indicated acceptor-containing protein constructs (measured using the LIFA method). Box plots show median, first and third quartile, with whiskers extending to the minimum and maximum and all individual data points that correspond to two independent experiments. **(c)** Representative images of various FRET pairs expressing COS-7 cells. Scale bar represents 5 µm. Source data are provided as Source Data file.

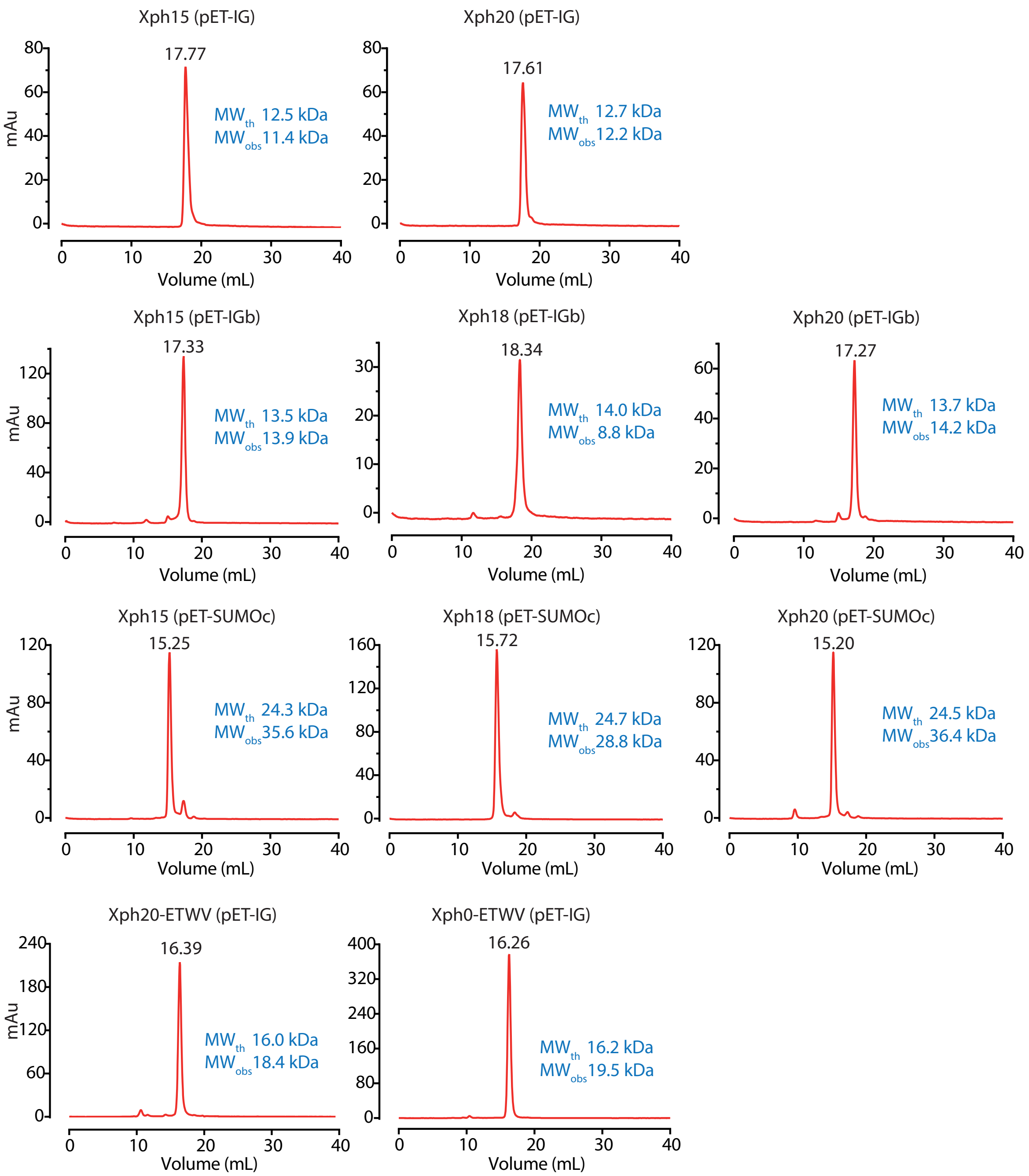

Supplementary Figure 6 | Size exclusion chromatograms of Xph15/18/20 constructs. The chromatograms were obtained on a HiLoad Superdex200 Increase 10/300 GL column with PBS + 0.01% Tween-20 as an eluent. Name of the clones are indicated with the bacterial vector used for expression in parenthesis and the elution volume. MW_{th} represents the theoretical molecular weight of the protein and MW_{obs} the molecular weight determined for the observed elution volumes after calibration of the column.

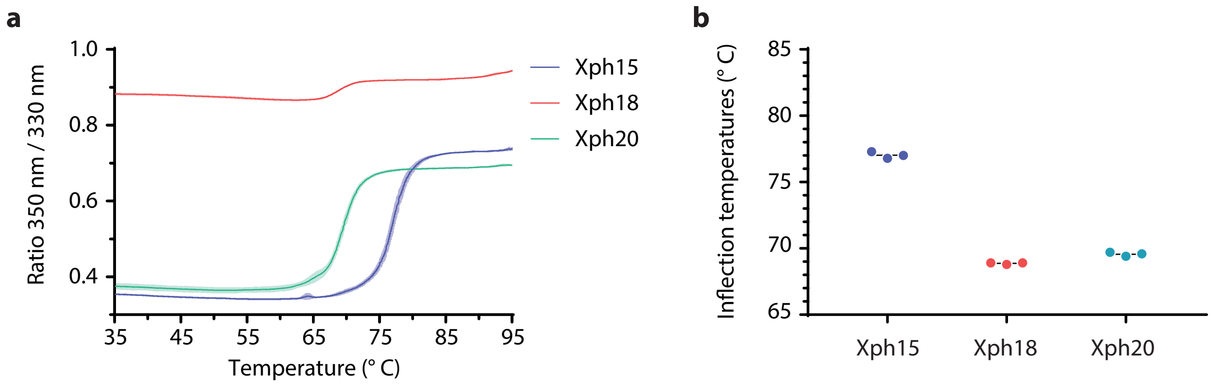

Supplementary Figure 7 | Stability of Xph15/18/20 evaluated on a Tycho NT.6 (NanoTemper Technologies) **(a)** Thermal unfolding curves (measurement of the variation of the fluorescence ratio of W and Y at 350 nm and 330 nm in function of temperature) for Xph15/18/20 samples with the S63K mutation at approximately 50 µM in PBS with 0.01% Tween-20 after storage at -80 °C. The data represent the mean of 3 runs with areas in lighter colors indicating the standard deviation. **(b)** Inflection temperatures of the unfolding transition (T_i) calculated by the instrument software from the thermal unfolding curves (for Xph15, T_i = 77.0 ± 0.3 °C; for Xph18, T_i = 68.9 ± 0.1 °C; for Xph20, T_i = 69.6 ± 0.2 °C, average ± s.d.).

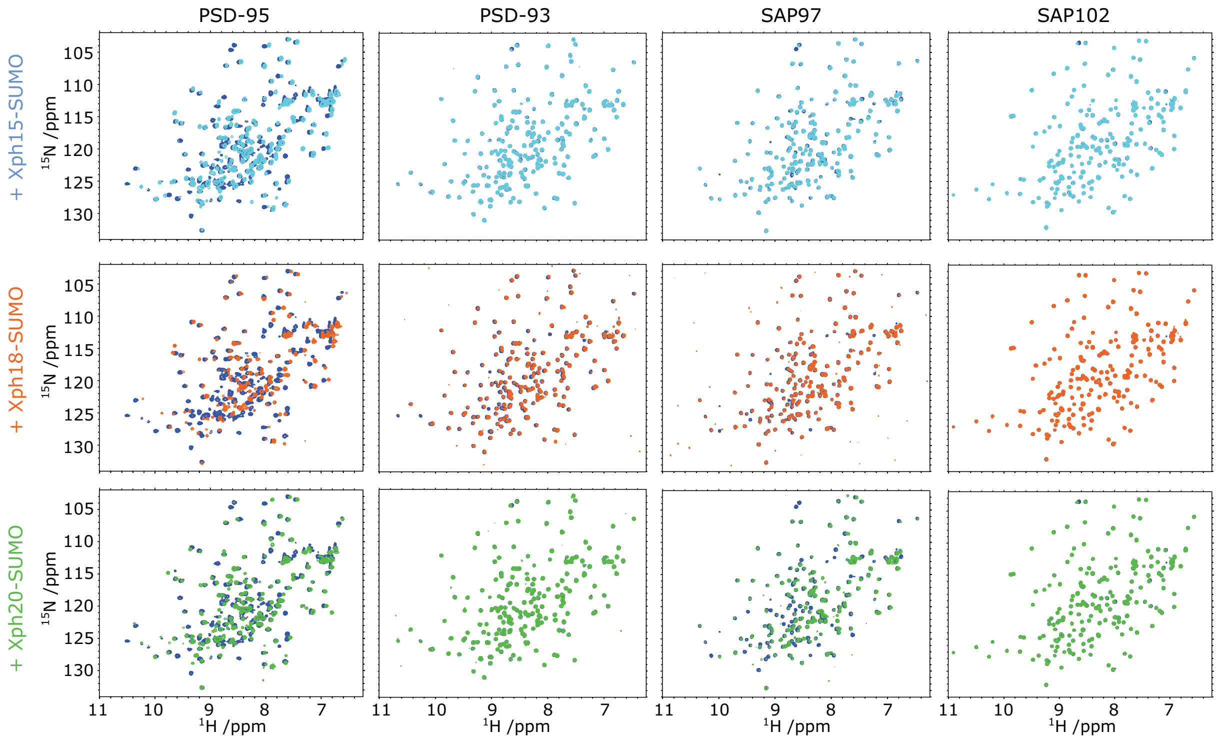

Supplementary Figure 8 | Binding specificity determined by NMR spectroscopy. Reference spectra (in blue) were collected on 80 μM ^{15}N-samples of PSD95-12, PSD93-12, SAP97-12, or SAP102-12 in PBS. Spectra were then collected after addition of 100 μM unlabeled Xph15 (cyan), Xph18 (orange) or Xph20 (green). All spectra were collected at 298 K.

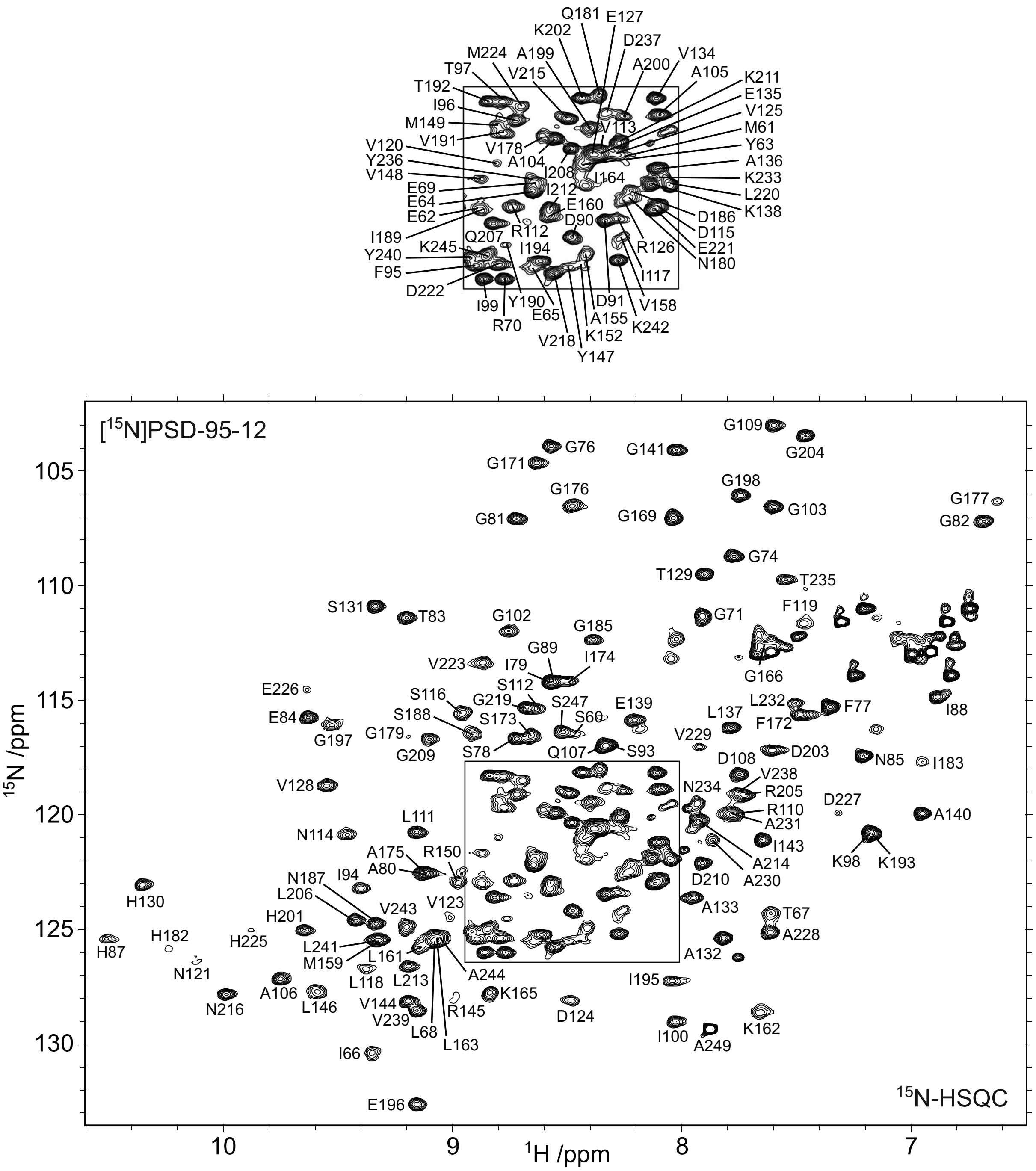

Supplementary Figure 9 | Annotated ^{15}N-HSQC of 80 µM [^{15}N]PSD-95-12 in PBS measured at 298 K and a field strength of 700 MHz. This spectrum is the reference spectrum in Fig. 3. Backbone amide crosspeaks have been annotated by residue type and number. The full list of backbone chemical shift assignments has been deposited in the Biological Magnetic Resonance Data Bank (BMRB) as entry 27308.

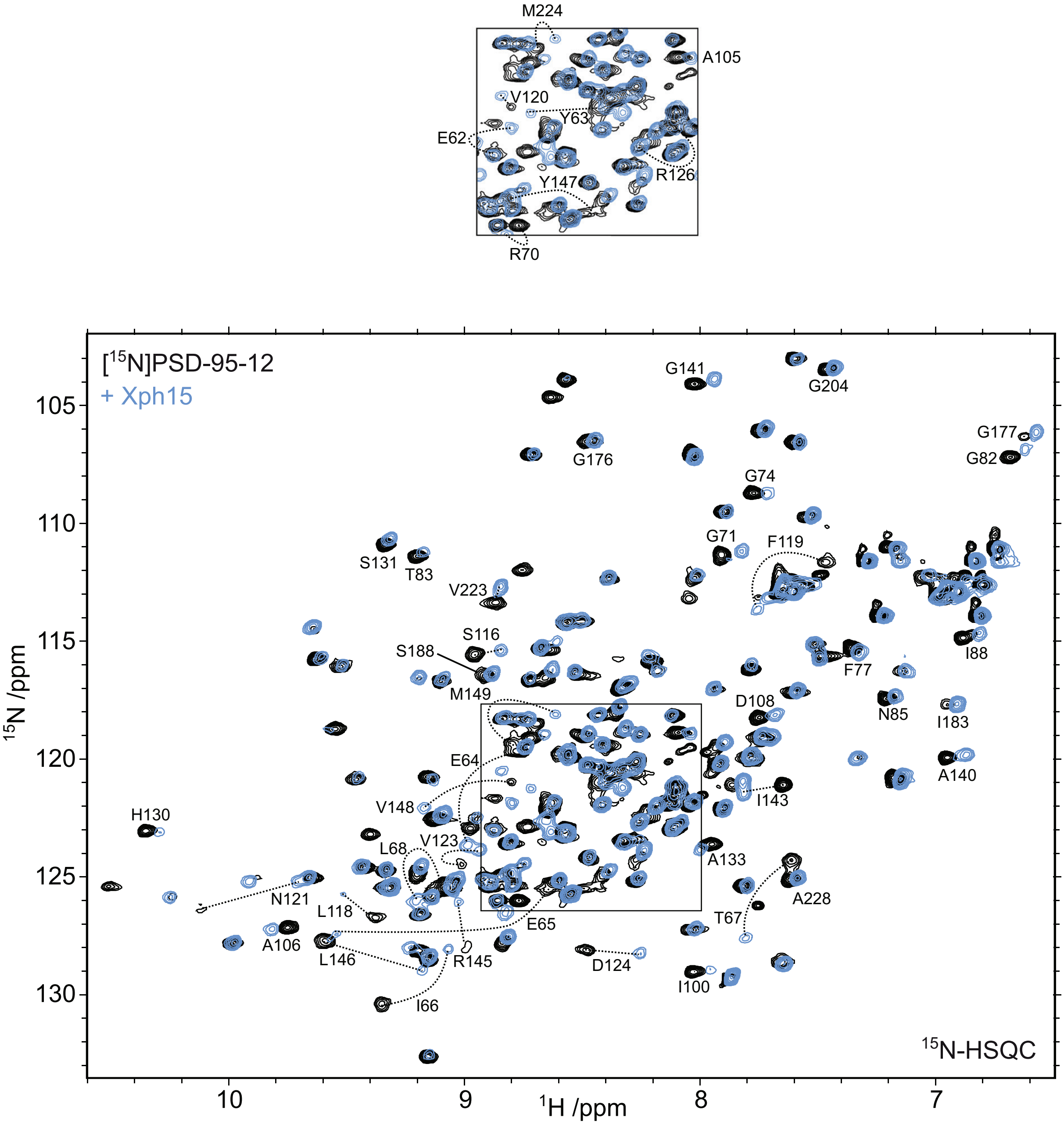

Supplementary Figure 10 | ^{15}N-HSQC spectrum of 80 µM [^{15}N]PSD-95-12 in the free form (black) and upon addition of 100 µM natural abundance Xph15, as presented in Fig. 3a. Spectra were collected in PBS at 298 K and a field strength of 700 MHz. Residue backbone amide crosspeaks that shift upon addition of Xph15 have been annotated by residue type and number, with the free and bound peak positions connected by a dotted line. Complete assignment of the reference spectrum is presented in Supplementary Fig. 9.

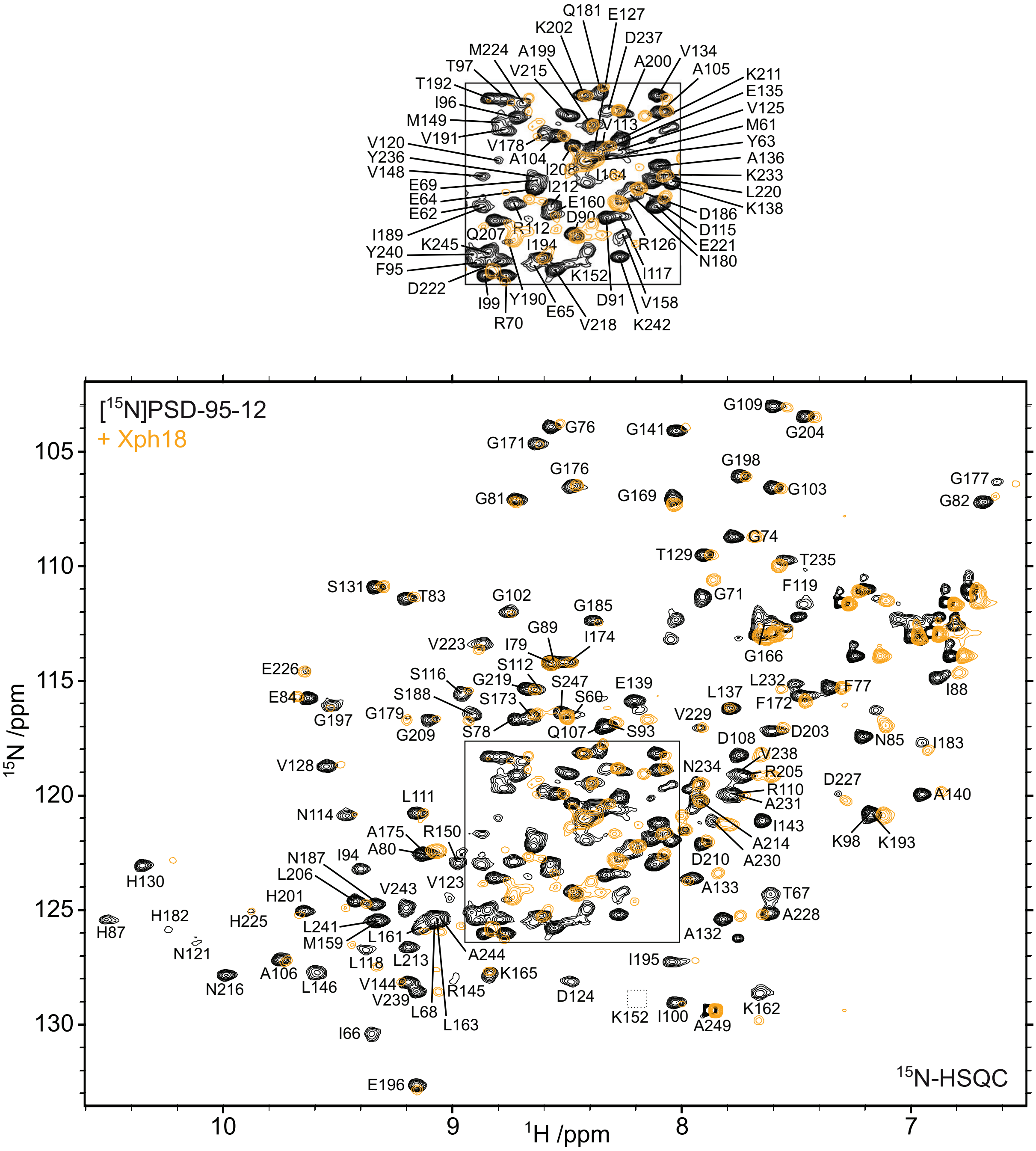

Supplementary Figure 11 | ^{15}N-HSQC spectrum of 80 µM [^{15}N]PSD-95-12 in the free form (black) and upon addition of 100 µM natural abundance Xph18, as presented in Fig. 3b. Spectra were collected in PBS at 298 K and a field strength of 700 MHz. Due to significant peak broadening upon addition of Xph18, all residue backbone amide crosspeaks of the free form have been annotated by residue type and number.

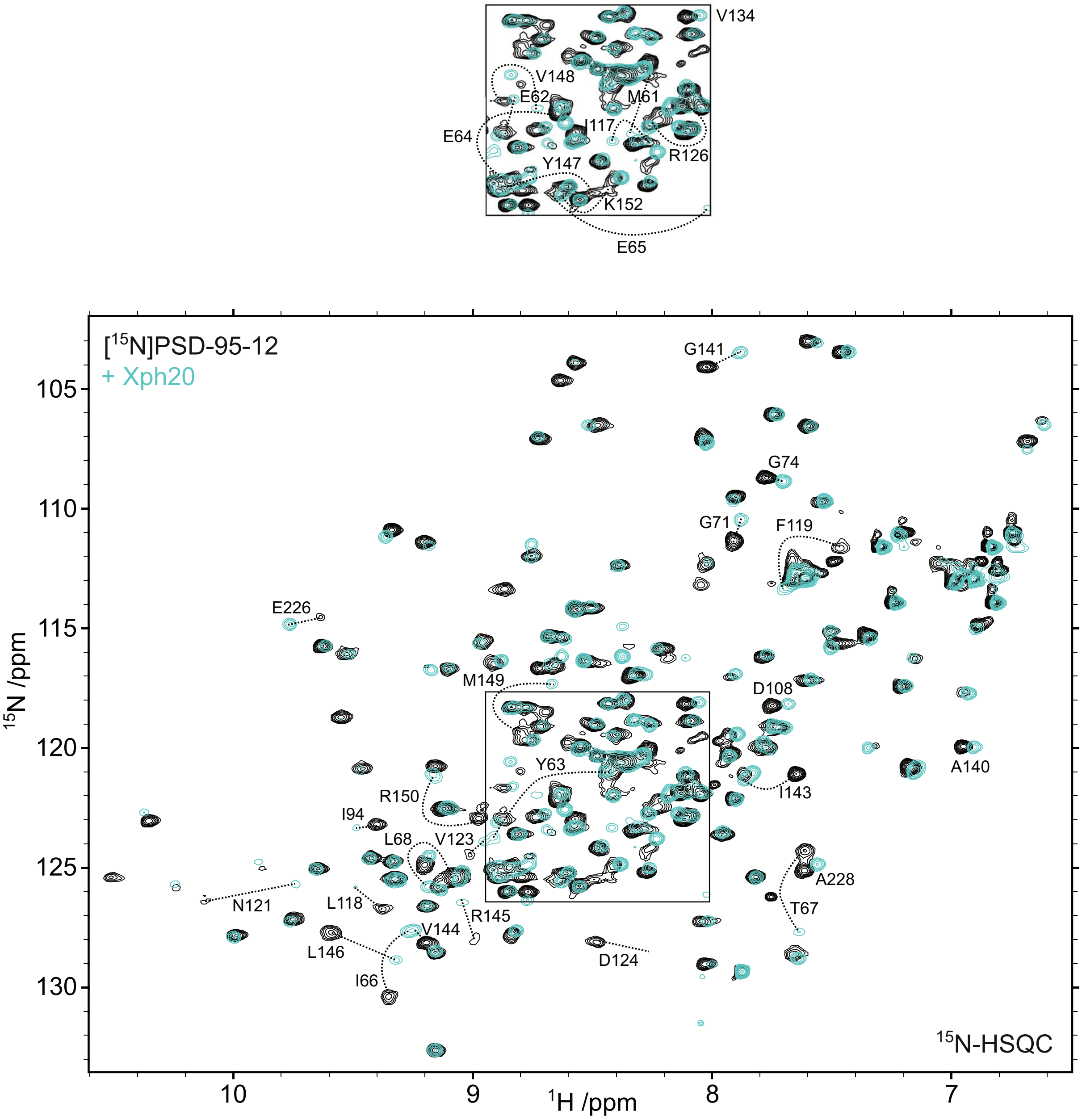

Supplementary Figure 12 | ^{15}N-HSQC spectrum of 80 µM [^{15}N]PSD-95-12 in the free form (black) and upon addition of 100 µM natural abundance Xph20, as presented in Fig. 3c. Spectra were collected in PBS at 298 K and a field strength of 700 MHz. Residue backbone amide crosspeaks that shift upon addition of Xph20 have been annotated by residue type and number, with the free and bound peak positions connected by a dotted line. Complete assignment of the reference spectrum is presented in Supplementary Fig. 9.

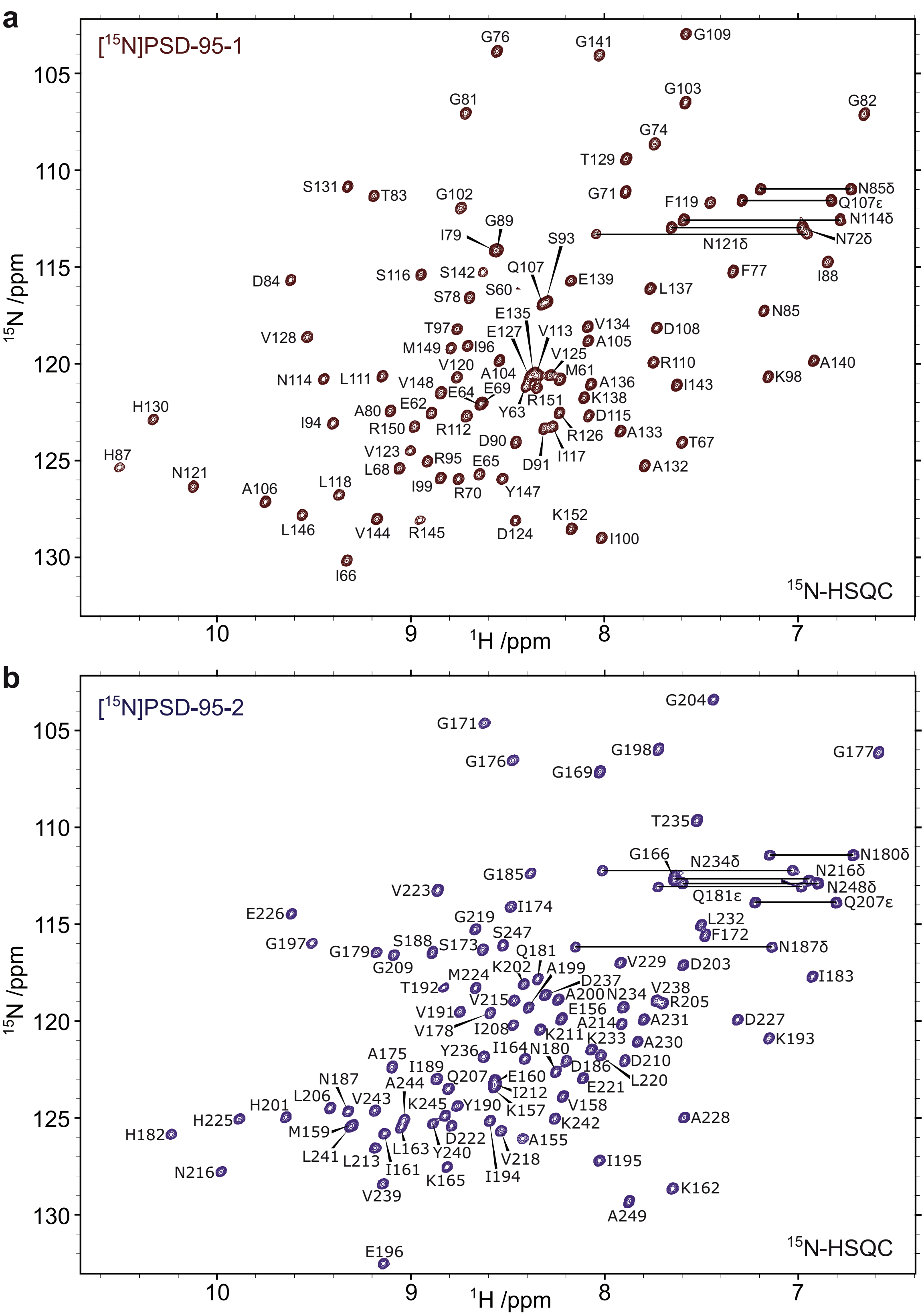

Supplementary Figure 13 | Annotated ^{15}N-HSQC of **(a)** 100 µM [^{15}N]PSD-95-1 and (**b**) 100 µM [^{15}N]PSD-95-2 in PBS measured at 298 K and a field strength of 700 MHz. These spectra were used as references in Supplementary Figs. 14-16. The full lists of backbone chemical shift assignments have been deposited in the Biological Magnetic Resonance Data Bank (BMRB) as entry 27309 for PSD-95-1 and entry 27310 for PSD-95-2.

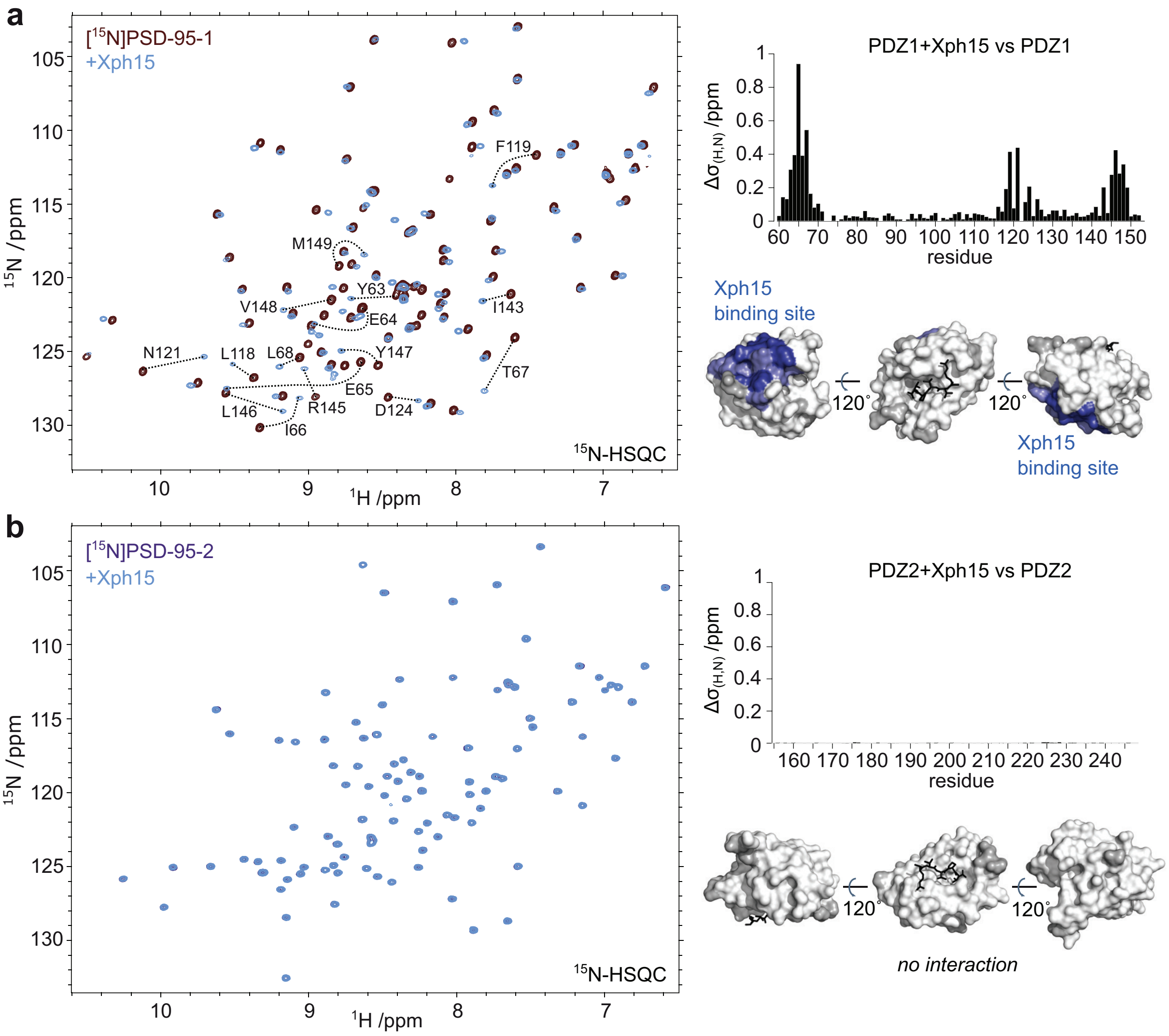

Supplementary Figure 14 | Xph15 binding to isolated PDZ domains of PSD-95. **(a)** ^{15}N-HSQC spectrum of 100 μM [^{15}N]PSD-95-1 in PBS for the free form (black) with overlay of the spectrum upon addition of 120 μM natural abundance Xph15 (blue). Spectra were collected at 298 K and a field strength of 700 MHz. Residue backbone amide crosspeaks that shift (>0.15 ppm) upon addition of Xph15 are annotated by residue type and number, and quantified by calculating the $\Delta\delta_{(H,N)}$ chemical shift perturbation (histogram). Residues with values greater than 0.15 have been colored blue on the surface representation of PSD-95-1. The PDZ1 domain is in the same orientation as in Figs 1 and 3. **(b)** A similar approach using PSD-95-2 demonstrates no changes to the PSD-95-2 spectrum upon addition of Xph15. Complete assignments of the PSD-95-1 and PDSD-95-2 reference spectra are presented in Supplementary Fig. 13. Source data are provided as Source Data file.

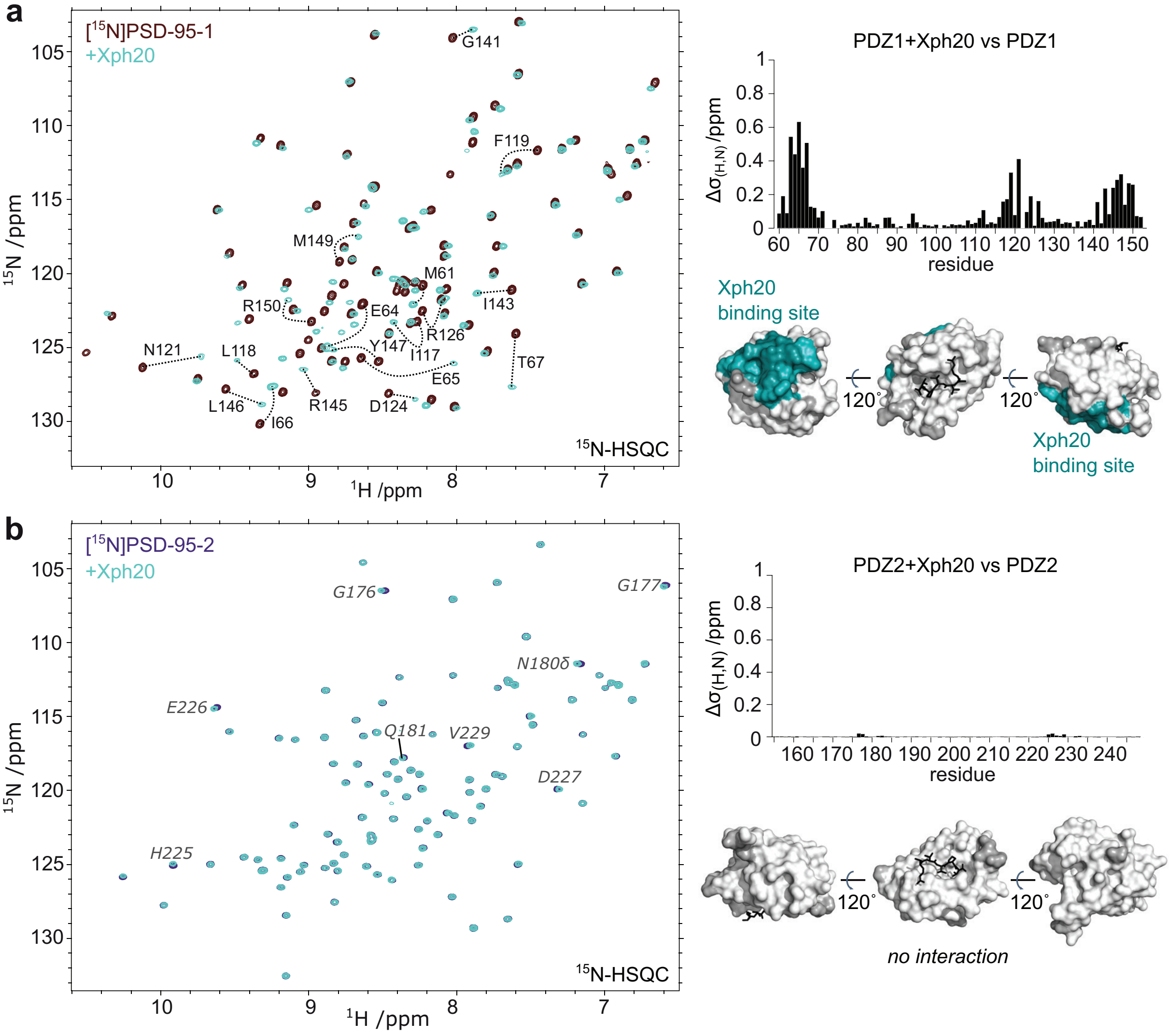

Supplementary Figure 15 | Xph20 binding to isolated PDZ domains of PSD-95. **(a)** ^{15}N-HSQC spectrum of 100 µM [^{15}N]PSD-95-1 in PBS for the free form (black) with overlay by the spectrum upon addition of 120 µM natural abundance Xph20 (teal). Spectra were collected at 298 K and a field strength of 700 MHz. Residue backbone amide crosspeaks that shift (>0.15 ppm) upon addition of Xph20 are annotated by residue type and number, and quantified by calculating the $\Delta\delta_{(H,N)}$ chemical shift perturbation (histogram). Residues with values greater than 0.15 have been colored teal on the surface representation of PSD-95-1. The PDZ1 domain is in the same orientation as in Figs 1 and 3. **(b)** A similar approach using PSD-95-2 demonstrates only minimal changes to the PSD-95-2 spectrum upon addition of Xph20 which have nevertheless been annotated. Complete assignments of the PSD-95-1 and PDSD-95-2 reference spectra are presented in Supplementary Fig. 13. Source data are provided as Source Data file.

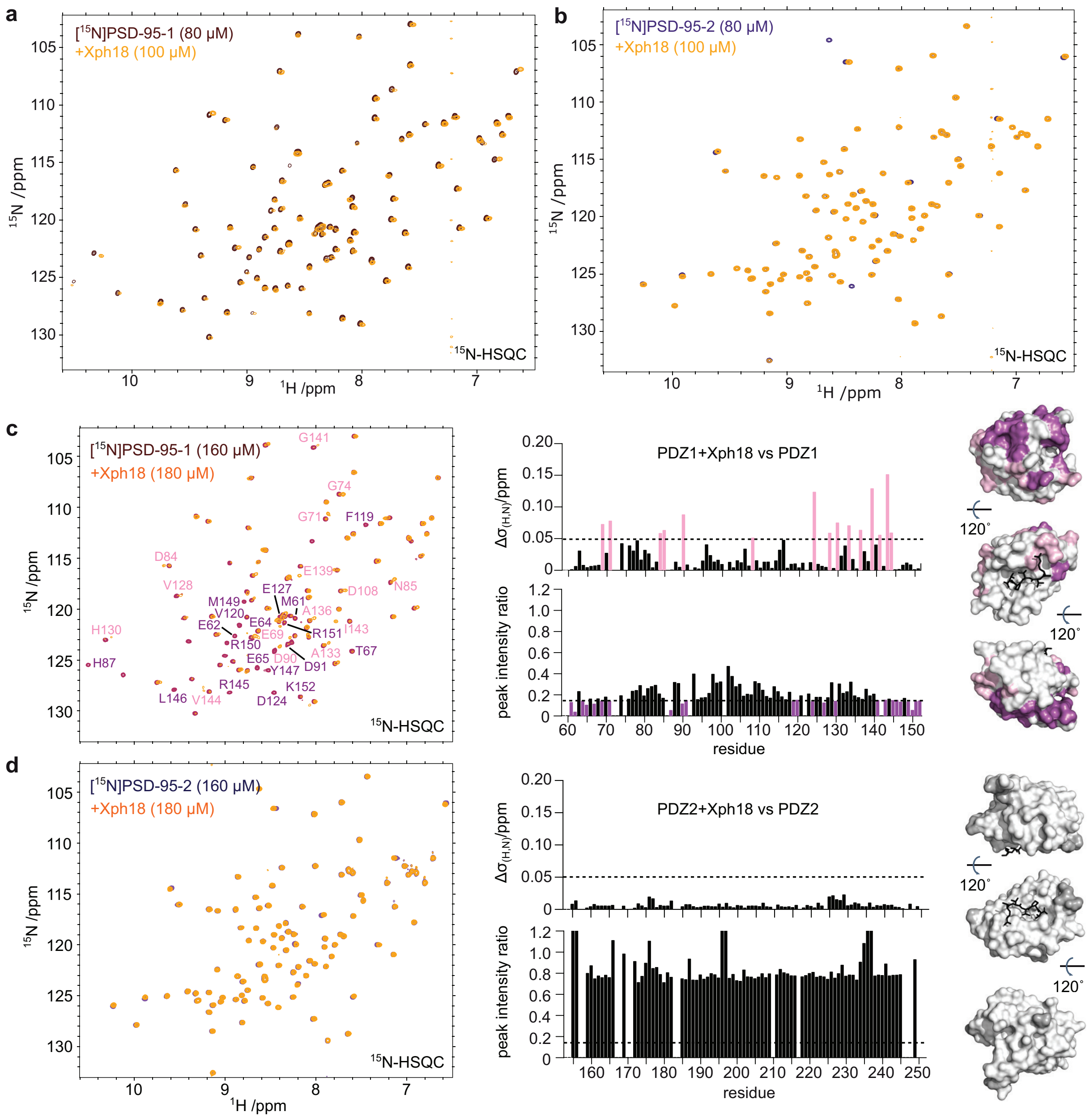

Supplementary Figure 16 | Xph18 binding to isolated PDZ domains of PSD-95. **(a,b)** Only minimal perturbation of residue backbone amide crosspeaks are observed upon addition of 120 µM natural abundance Xph18 to either **(a)** 100 µM [^{15}N]PSD-95-1 or **(b)** 100 µM [^{15}N]PSD-95-2. The spectra in black corresponds to the ^{15}N-labelled free form, and in orange following addition of Xph18. Spectra were collected at 298 K and a field strength of 700 MHz. **(c,d)** At higher protein concentrations, only backbone amide crosspeaks from [^{15}N]PSD-95-1 shift or are broadened. **(c)** Amide crosspeaks that shift by greater than 0.05 ppm (pink) or have a peak intensity ratio (intensity of the bound crosspeak divided by the intensity of the unbound crosspeak) below 0.15 (purple) are annotated in the ^{15}N-HSQC, and colored in the corresponding histograms and surface representations. Note that the chemical shift perturbation histogram has a different scale than for all of the previous histograms, and in addition the lowered threshold of 0.05 pm reflects a reduced range of perturbation. Complete assignments of the PSD-95-1 and PDSD-95-2 reference spectra are presented in Supplementary Fig. 13. Source data are provided as Source Data file.

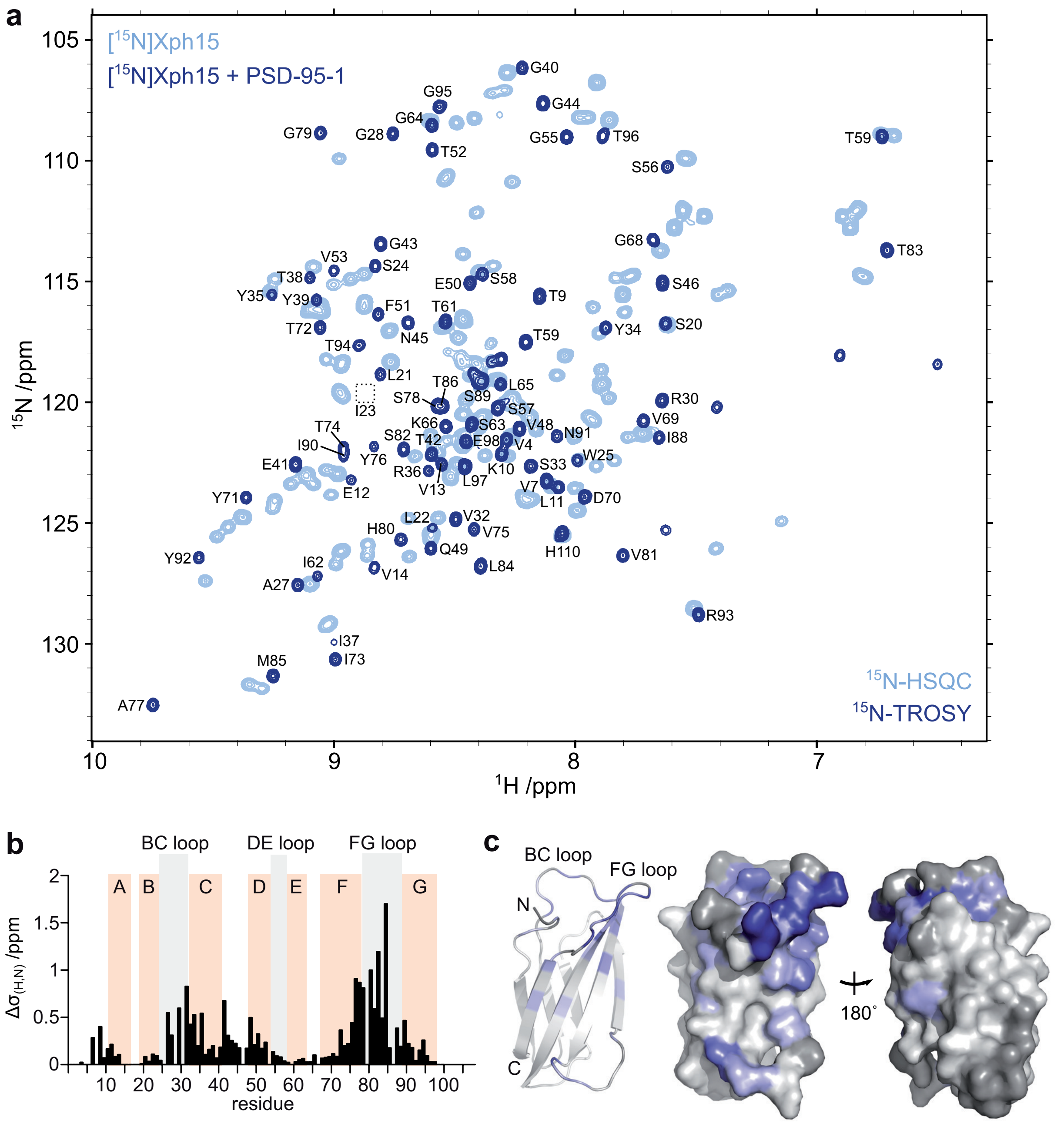

Supplementary Figure 17 | PSD-95-1 binding site on Xph15. (a) ^{15}N-HSQC spectrum of the free from of [^{15}N]Xph15 (light blue) with overlay of the ^{15}N-TROSY spectrum of [^{15}N]Xph15 following addition of 1.2 molar equivalents of natural abundance PSD-95-1 (dark blue). The residue backbone amide crosspeaks for the bound form of Xph15 are annotated with residue type and number. Annotation of the free form spectrum of Xph15 is included in Supplementary Figure 19. **(b)** Quantification of chemical shift perturbation $\Delta\delta_{(H,N)}$ between the free Xph15 and in complex with PDS-95-1. The location of the BC, DE and FG loop residues are indicated. **(c)** Residues with $\Delta\delta_{(H,N)}$ values greater than 0.25 have been colored blue on cartoon and surface representations of the Xph15 structure (see Methods for details on the SWISS-MODEL homology modelling). Source data are provided as Source Data file.

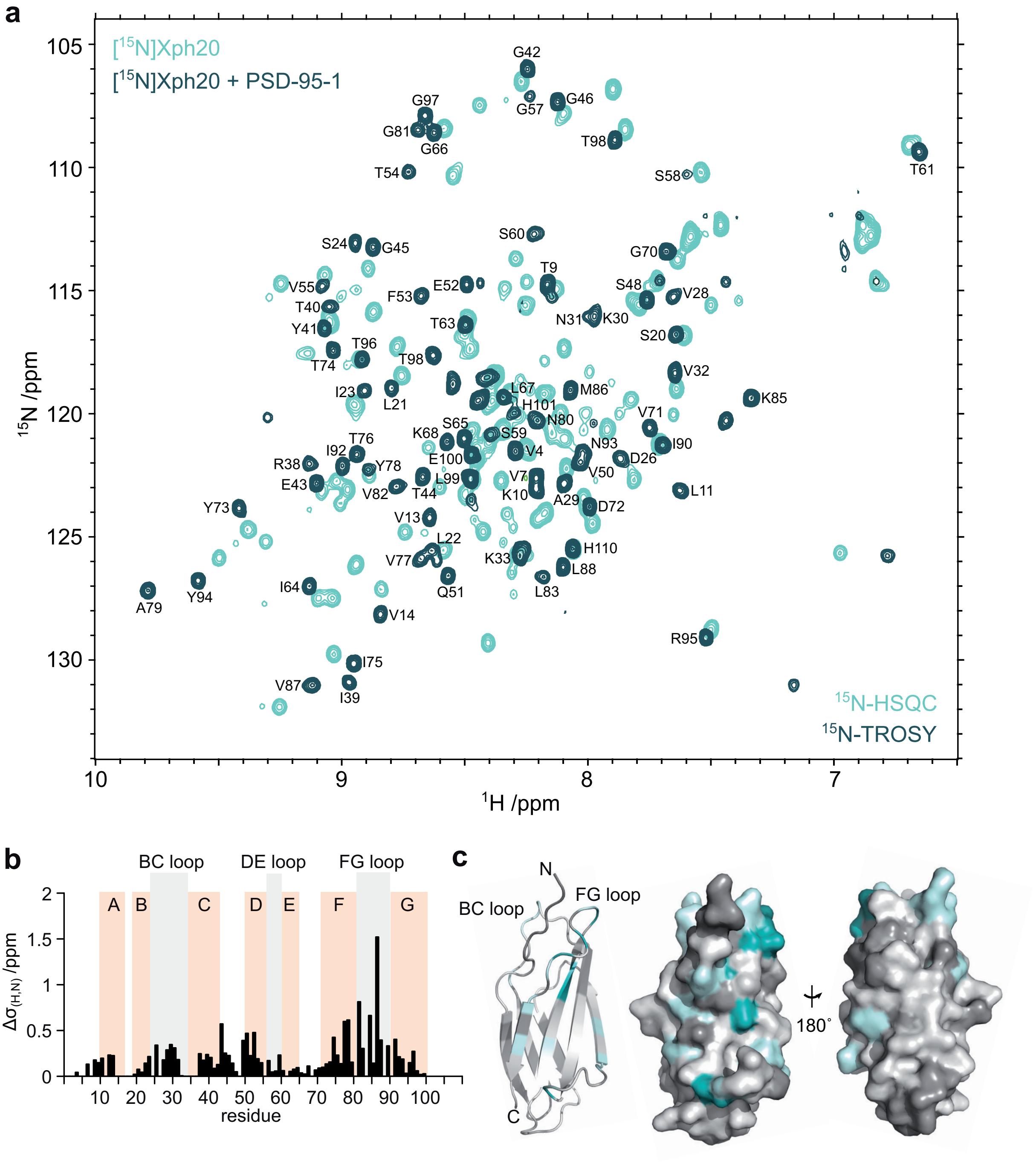

Supplementary Figure 18 | PSD-95-1 binding site on Xph20. **(a)** ^{15}N-HSQC spectrum of the free from of [^{15}N]Xph20 (light teal) with overlay of the ^{15}N-TROSY spectrum of [^{15}N]Xph20 following addition of 1.2 molar equivalents of natural abundance PSD-95-1 (dark teal). The residue backbone amide crosspeaks for the bound form of Xph20 are annotated with residue type and number. Annotation of the free form spectrum of Xph20 is included in Supplementary Figure 20. **(b)** Quantification of chemical shift perturbation $\Delta\delta_{(H,N)}$ between the free Xph20 and in complex with PDS-95-1. The location of the BC, DE and FG loop residues are indicated. **(c)** Residues with $\Delta\delta_{(H,N)}$ values greater than 0.25 have been colored teal on cartoon and surface representations of the Xph20 structure (see Methods for details on the SWISS-MODEL homology modelling). Source data are provided as Source Data file.

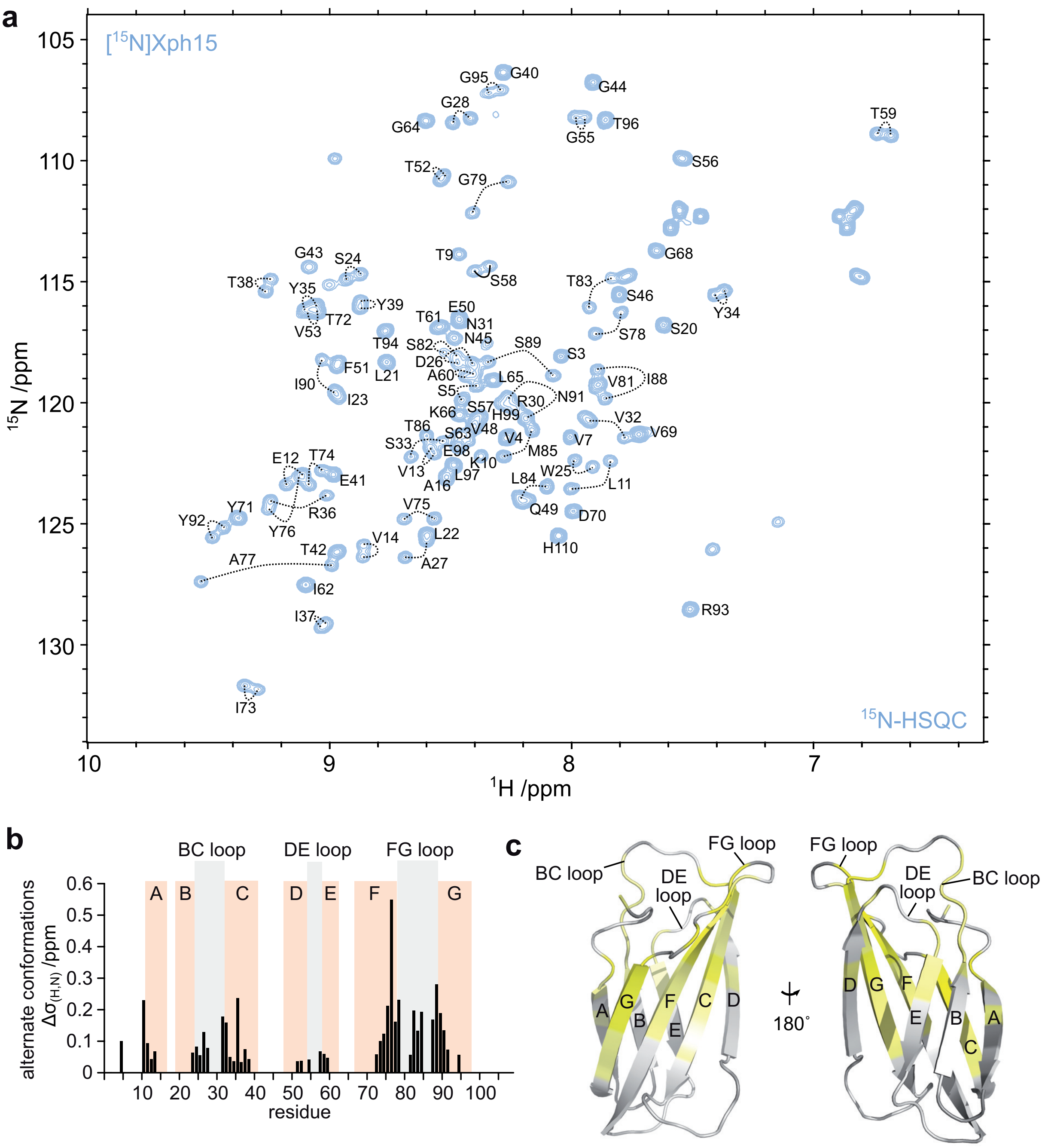

Supplementary Figure 19 | Two equal conformational populations for free Xph15. **(a)** ^{15}N-HSQC spectrum of the free from of [^{15}N]Xph15 with residue backbone amide crosspeaks annotated with residue type and number. Crosspeaks corresponding to the same residue are connected by a dotted line. **(b)** Quantification of chemical shift perturbation $\Delta\delta_{(H,N)}$ between the two population of free Xph15. The location of the BC, DE and FG loop residues are indicated. **(c)** All residues displaying two populations have been colored yellow on cartoon and surface representations of the Xph15 structure (see Methods for details on the SWISS-MODEL homology modelling). The individual β-strands have been labelled A-G. Source data are provided as Source Data file.

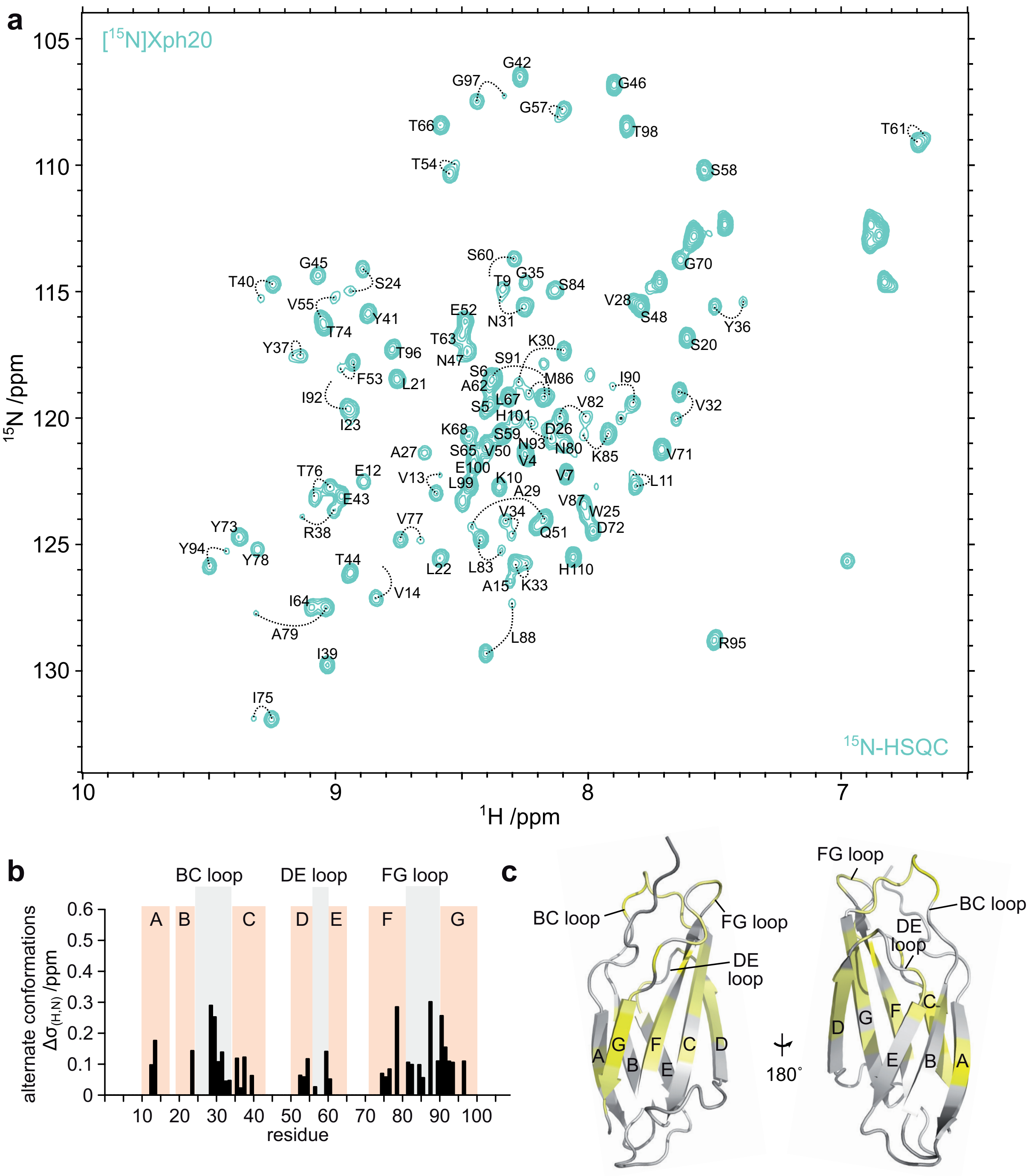

Supplementary Figure 20 | Major and minor conformational populations for free Xph20. (a) ^{15}N-HSQC spectrum of the free from of [^{15}N]Xph20 with residue backbone amide crosspeaks annotated with residue type and number. Crosspeaks corresponding to the same residue are connected by a dotted line. **(b)** Quantification of chemical shift perturbation $\Delta\delta_{(H,N)}$ between the major and minor populations of free Xph20. The location of the BC, DE and FG loop residues are indicated. **(c)** All residues displaying two populations have been colored yellow on cartoon and surface representations of the Xph20 (see Methods for details on the SWISS-MODEL homology modelling). The individual β-strands have been labelled A-G. Source data are provided as Source Data file.

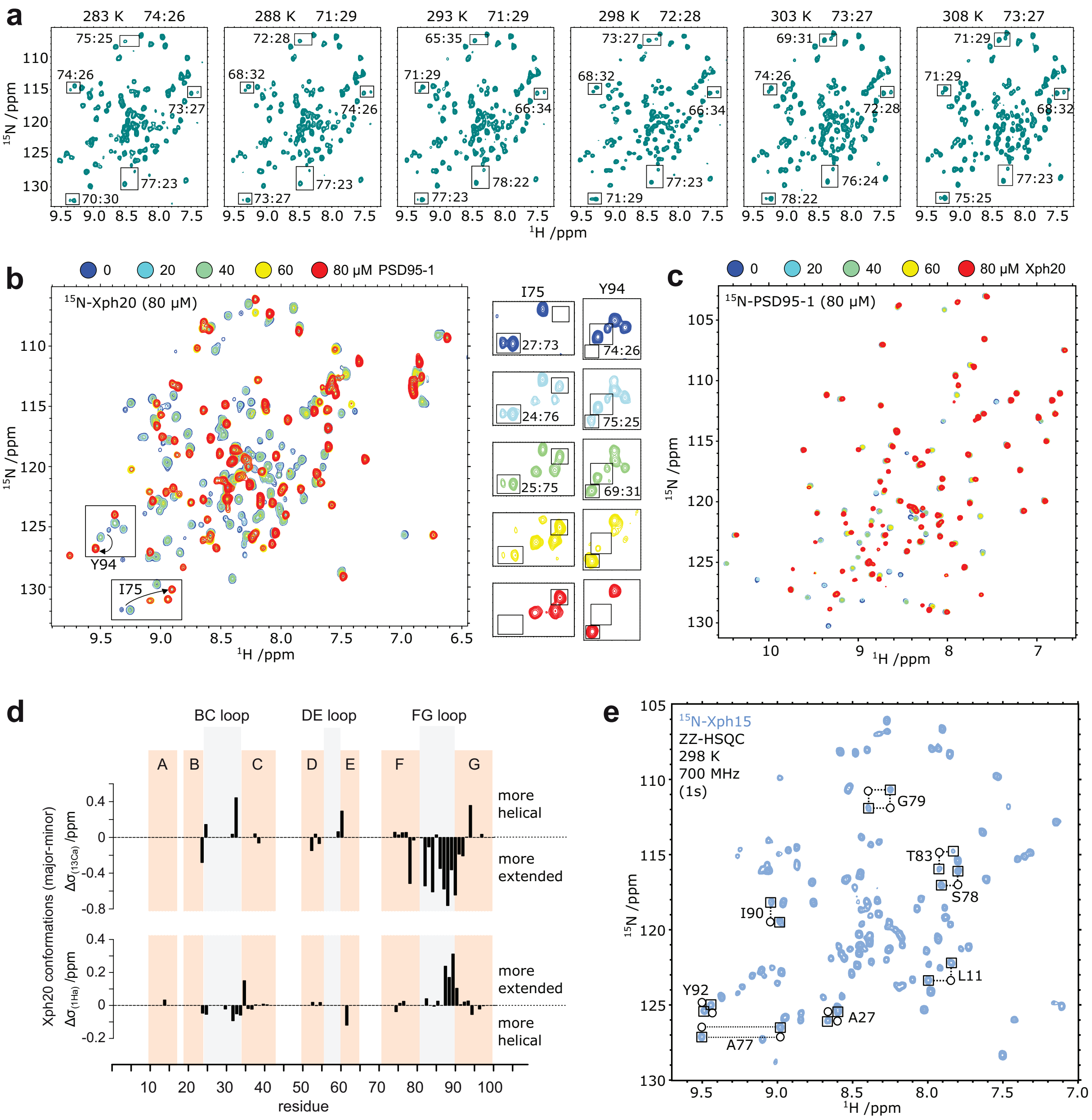

Supplementary Figure 21 | Characterization of the two populations of Xph15 and Xph20. **(a)** ^{1}H,^{15}N-HSQC spectra collected on unbound 80 μM [^{15}N]Xph20 at temperatures of 283, 288, 293, 298, 303 and 308 K. The crosspeak intensity ratios for the major and minor conformation for five residues are indicated within the spectra, and the average of the five values indicated above each spectrum. No change in the ratio is observed in this temperature range. **(b)** Titration of 80 μM [^{15}N]Xph20 with unlabeled PSD-95-1. Crosspeak intensity ratios of the major and minor conformations for I75 and Y94 are indicated for each titration point, which show a consistent ratio throughout the titration. Note that only one conformation is found in the bound form. **(c)** Titration of 80 μM [^{15}N]PSD-95-1 with unlabeled Xph20, confirming that only one bound conformation is observed during the titration. **(d)** Difference in ^{1}Hα and ^{13}Cα chemical shifts between the major and minor populations of Xph20. These secondary chemical shifts (Δδ) suggest the β-strand G may extend further into the FG loop for the major population. **(e)** ^{1}H^{15}N-ZZ-HSQC to measure the rate of exchange between the two equal populations of Xph15. No exchange peaks are observed for a 1 s mixing time (and also for 500 ms and 2 s, not shown).

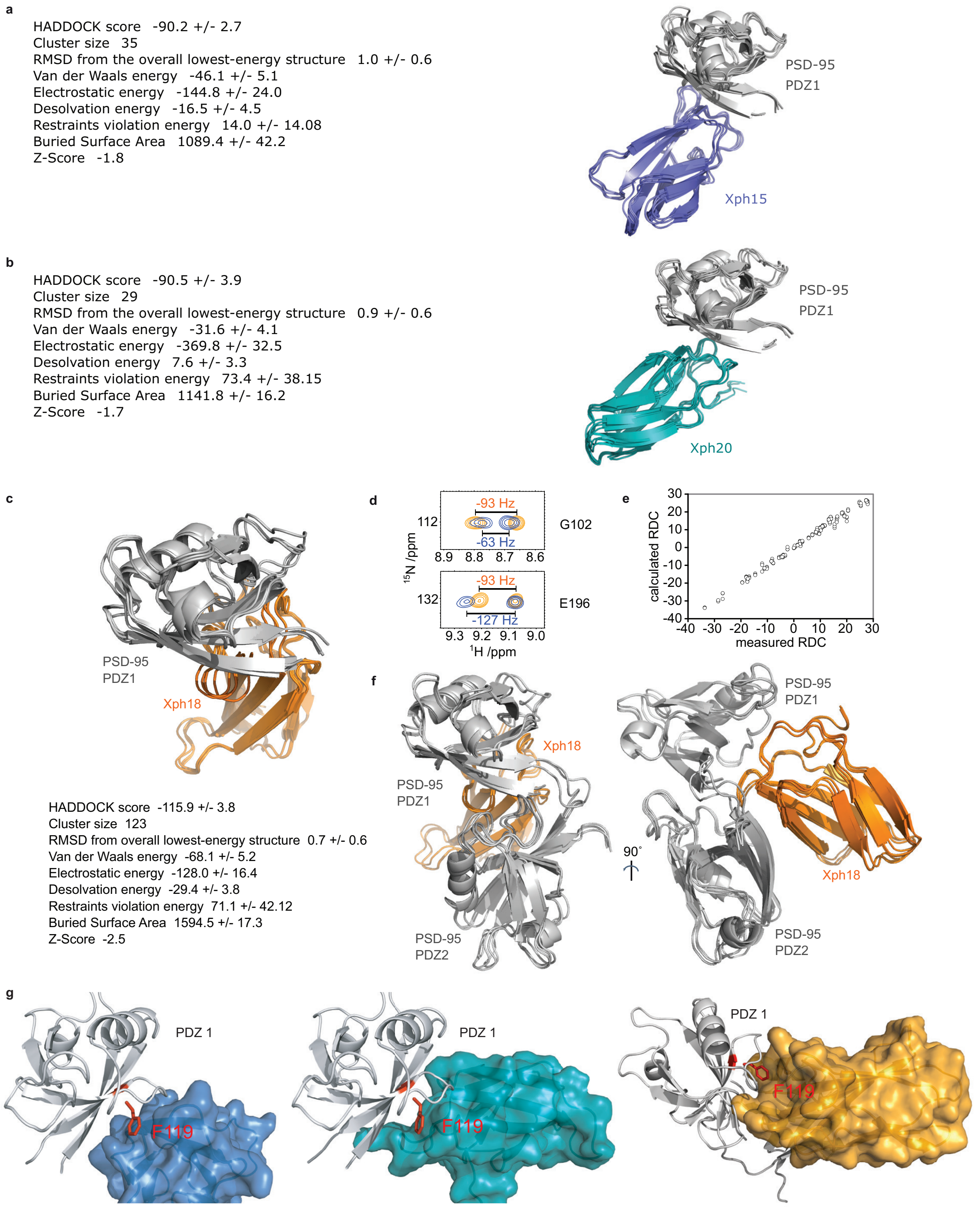

Supplementary Figure 22 | Docking models for PSD-95 PDZ domain1 complexes with **(a)** Xph15, **(b)** Xph20 and **(c)** Xph18. Statistics related to the top cluster of structures are included (mean ± s.d.). For details of the parameters see Methods. For each complex the four lowest energy models have been aligned and illustrated by cartoon representation. **(d)** Representative splitting in the ^{1}H,^{15}N backbone amide crosspeaks for isotropic (black) and anisotropic (orange) [70%-^{2}H,^{15}N]PSD-95-12 bound to unlabeled Xph18. **(e)** Back calculation of ^{1}H,^{15}N residual dipolar coupling (RDC) in relation to calculated values, by using MODULE2. **(f)** Combined RDC and HADDOCK model for PSD-95-12 in complex with Xph18. **(g)** Molecular details of the interaction between PDZ domain 1 and Xph15 (left, blue), Xph20 (middle, teal) or Xph18 (right, orange) with PDZ domain 1 F119 side chain highlighted in red.

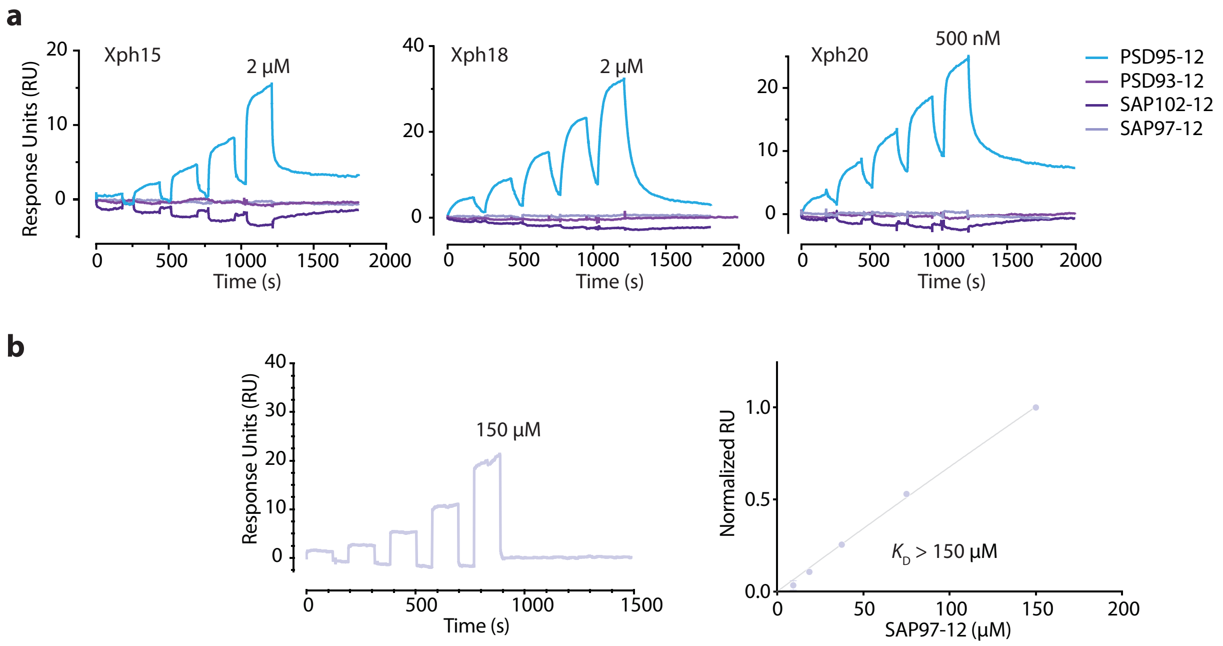

Supplementary Figure 23 | (a) Representative SPR sensorgrams obtained on a Biacore X100 by single cycle kinetics of Xph15/18/20 (analyte) against immobilized biotinylated tandem PDZ domains (ligand). The reported concentrations represent the highest concentration used on the final analyte injection, the previous 4 injections are a series of two-fold dilutions. For each experiment, the ligand density was kept in a similar range (around 100 RU for PSD-95 and > 100 for the other tandems) to allow direct comparison. The curves obtained in that experimental configuration with PSD-95 all showed a biphasic behavior preventing a quantitative kinetic analysis. The qualitative results are nonetheless consistent with experiments performed with the reverse configuration. Xph15/18/20 show potent binding to PSD-95 and no significant binding was observed for SAP97, SAP102 and PSD-93. **(b)** Left, representative SPR sensorgram obtained on a Biacore T200 by single cycle kinetics of SAP97-12 (analyte) against immobilized biotinylated Xph20 (ligand). The reported concentrations represent the highest concentration used on the final analyte injection, the previous 4 injections are a series of two-fold dilutions. Right, tentative equilibrium analysis of the sensorgrams obtained for SAP97-12. Each data point represents the average of three independent measurements ± s.d. The data points were fitted with the one site binding (hyperbola) equation from GraphPad Prism 7.04. Source data are provided as Source Data file.

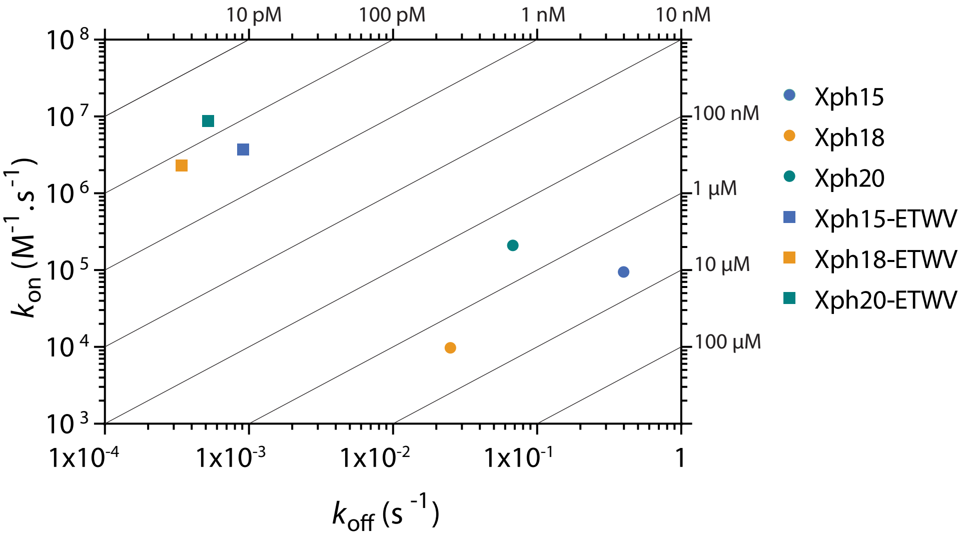

Supplementary Figure 24 | Rate Plane with Isoaffinity Diagonals (RaPID) plot for SPR data obtained in main Fig 5 and 6. The plot illustrates how different pairs of rate constants (association, k_{on}, and dissociation, k_{off}), resulting in different sensorgram shapes, will ultimately lead to similar dissociation constants (K_D). It is interesting to note that the relative differences in rate constants between the isolated clones are conserved in the resulting fusion constructs.

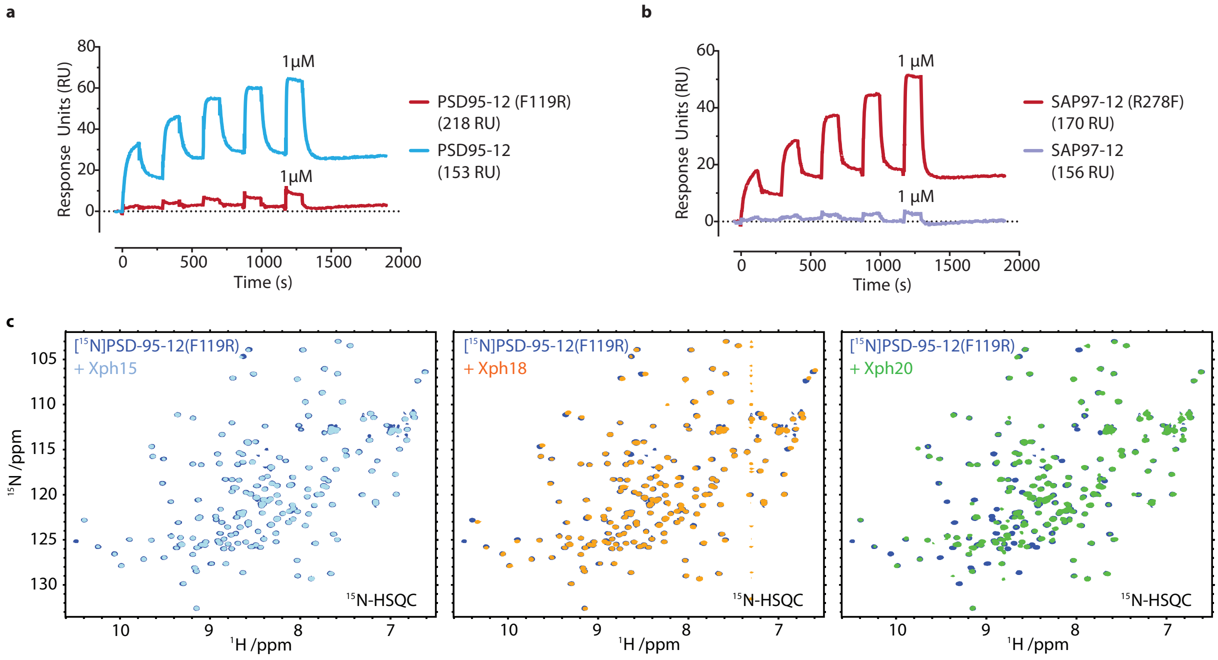

Supplementary Figure 25 | (a-b) Representative SPR sensorgrams obtained on a Biacore X100 by single cycle kinetics of Xph20 (S63K mutant, analyte) against indicated biotinylated tandem PDZ domains (ligand). The reported concentrations represent the highest concentration used on the final analyte injection, the previous 4 injections are a series of two-fold dilutions. For each experiment, the ligand density was kept in a similar range as indicated in parenthesis in the legends to allow direct comparison. The curves obtained in that experimental configuration with PSD-95-12 and SAP97-12 (R278F) present a biphasic behavior preventing a quantitative kinetic analysis. **(a)** Binding of Xph20 to PSD-95-12 wild type or F119R mutant. The wild type shows strong binding to Xph20 while in the same conditions the F119R mutant shows negligible binding. **(b)** Binding of Xph20 to SAP97-12 wild type or R287F mutant. The wild type shows negligible binding to Xph20 (comparable to PSD-95 F119R mutant) while in the same conditions the R287F mutant shows strong binding comparable to that of wild-type PSD-95-12. **(c)** NMR reference spectrum of the unbound F119R mutant of PSD-95-12 (in blue) overlaid with spectra following addition of Xph15 (light blue), Xph18 (orange) or Xph20 (green). The F119R mutant of PSD-95-12 shows decreased binding to all three clones as compared to wildtype PSD-95-12, as evident by comparison to the spectra in Fig. 3 and Supplementary Figs. 8, 10-12.

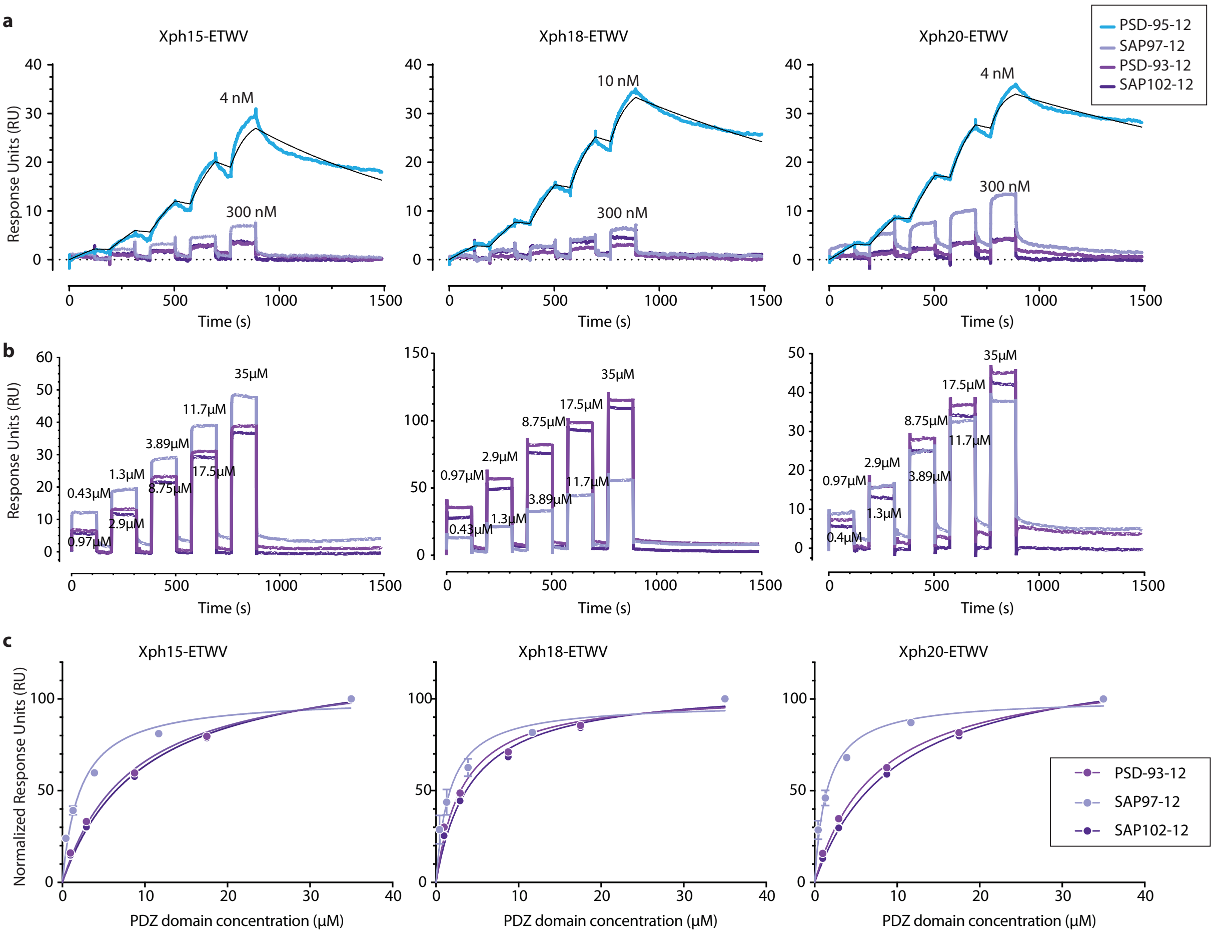

Supplementary Figure 26 | (a) Representative SPR sensorgrams obtained on a Biacore T200 by single cycle kinetics of tandem PDZ domains (analyte) against immobilized biotinylated Xph15/18/20-ETWV (ligand). The reported concentrations represent the highest concentration used on the final analyte injection, the previous 4 injections are a series of two-fold dilutions. For each experiment, the ligand density was kept in a similar range to allow direct comparison. The colored curves represent measured data points and black lines represent the global fit obtained with a 1:1 binding model used for analysis. **(b)** Representative SPR sensorgrams obtained on a Biacore T200 by single cycle kinetics of selected tandem PDZ domains (analyte) at higher concentrations against immobilized biotinylated Xph15/18/20-ETWV (ligand). **(c)** Equilibrium analysis of the sensorgrams obtained in **b** for SAP97-12, SAP102-12 and PSD-93-12 (values normalized to the highest response). Each data point represents the mean of two independent measurements ± s.d. The data points were fitted with the one site binding (hyperbola) equation from GraphPad Prism 7.04. Dissociation constants are reported in Supplementary table S1. Source data are provided as Source Data file.

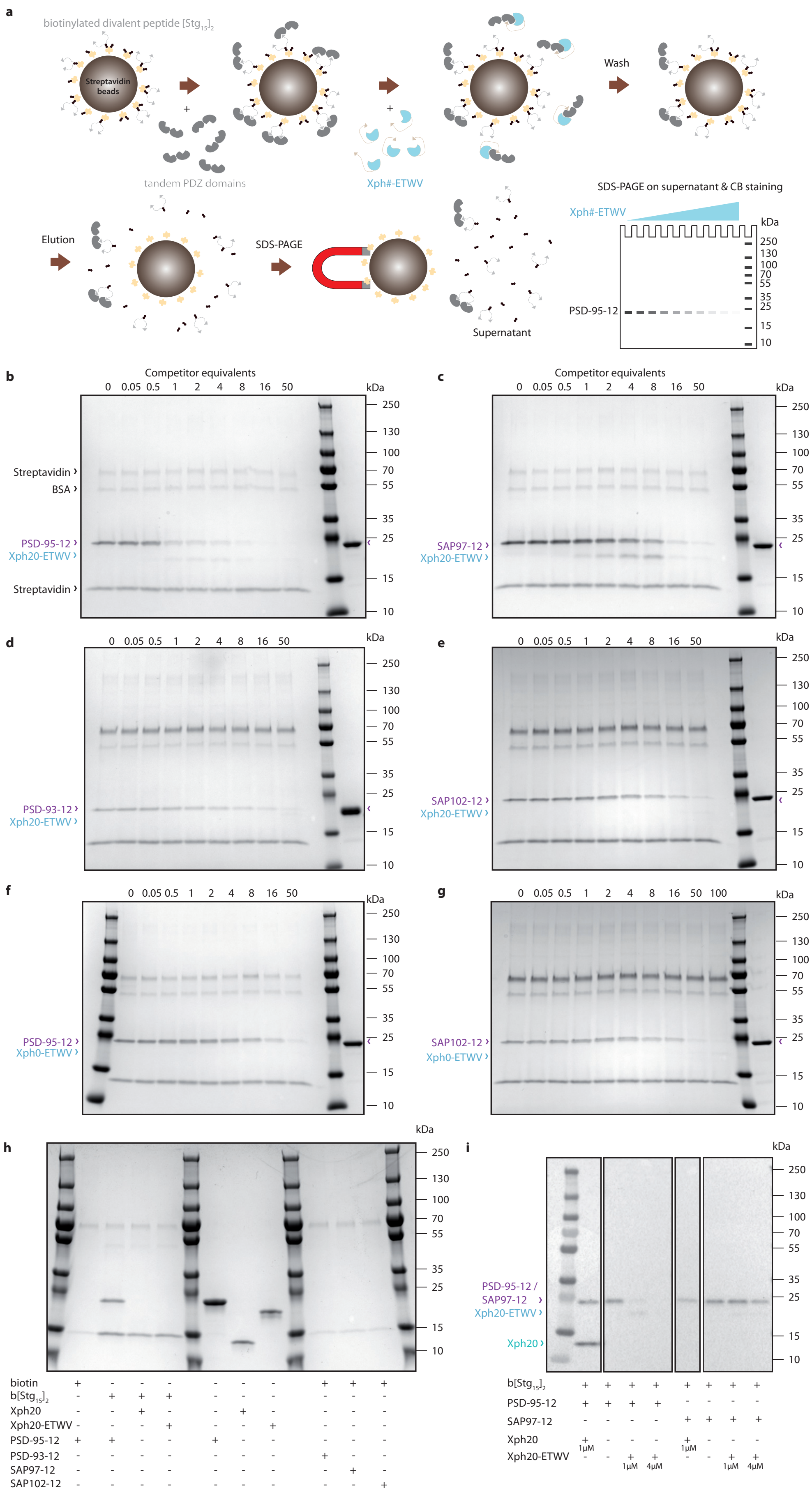
a
biotinylated divalent peptide [Stg15]2
Streptavidin beads
tandem PDZ domains
Xph#-ETWV
Wash
Elution
SDS-PAGE
Supernatant
SDS-PAGE on supernatant & CB staining
kDa
250
130
100
70
55
35
25
15
10
PSD-95-12
b
Competitor equivalents
0 0.05 0.5 1 2 4 8 16 50
Streptavidin
BSA
PSD-95-12
Xph20-ETWV
Streptavidin
c
SAP97-12
Xph20-ETWV
d
PSD-93-12
Xph20-ETWV
e
SAP102-12
Xph20-ETWV
f
PSD-95-12
Xph0-ETWV
g
0 0.05 0.5 1 2 4 8 16 50 100
SAP102-12
Xph0-ETWV
h
biotin
b[Stg15]2
Xph20
Xph20-ETWV
PSD-95-12
PSD-93-12
SAP97-12
SAP102-12
i
PSD-95-12 /
SAP97-12
Xph20-ETWV
Xph20
b[Stg15]2
PSD-95-12
SAP97-12
Xph20
Xph20-ETWV
1µM
4µM

Supplementary Figure 27 | Competitive titrations of Xph20-ETWV against tandem PDZ domains and divalent ligand. **(a)** Competition scheme. Streptavidin-coated magnetic beads are first functionalized with biotinylated divalent ligands (derived from the last 15 residues of stargazin) then incubated with tandem PDZ domains. The resulting complexes on beads are next titrated with various amounts of Xph20-ETWV (or Xph0-ETWV as a control). After several washes, the material left on the beads is eluted by heating the beads at 75 ºC in sample loading buffer and analyzed by SDS-PAGE. **(b-g)** Representative SDS-PAGE analysis (Colloidal blue stain, uncropped gels Fig 6c) of pull-down material consecutive to the competitive titration. Dark arrows indicate the streptavidin monomers and tetramers from the beads and the BSA used to coat the beads and prevent non-specific interactions. Purple arrows indicate the tandem PDZ domains (in each gel, the tandem PDZ domains used for the corresponding experiment was deposited in the rightmost lane). Light blue arrows indicate Xph20- or Xph0-ETWV. **(b)** Gel obtained for Xph20-ETWV and PSD-95-12. **(c)** Gel obtained for Xph20-ETWV and SAP97-12. **(d)** Gel obtained for Xph20-ETWV and PSD-93-12. **(e)** Gel obtained for Xph20-ETWV and SAP102-12. **(f)** Gel obtained for Xph0-ETWV and PSD-95-12. **(g)** Gel obtained for Xph0-ETWV and SAP102-12. **(h)** Control gel (Colloidal blue stain) for the absence of non-specific binding of PSD95-12 and all the other tandem DLG PDZ domains in the absence of biotinylated divalent peptide as well as of Xph20-ETWV or Xph20 in the absence of PSD-95 PDZ domains. **(i)** Western blot analysis (HisProbe HRP) of pull-down material in presence of PSD-95 or SAP97 tandem PDZ domains.

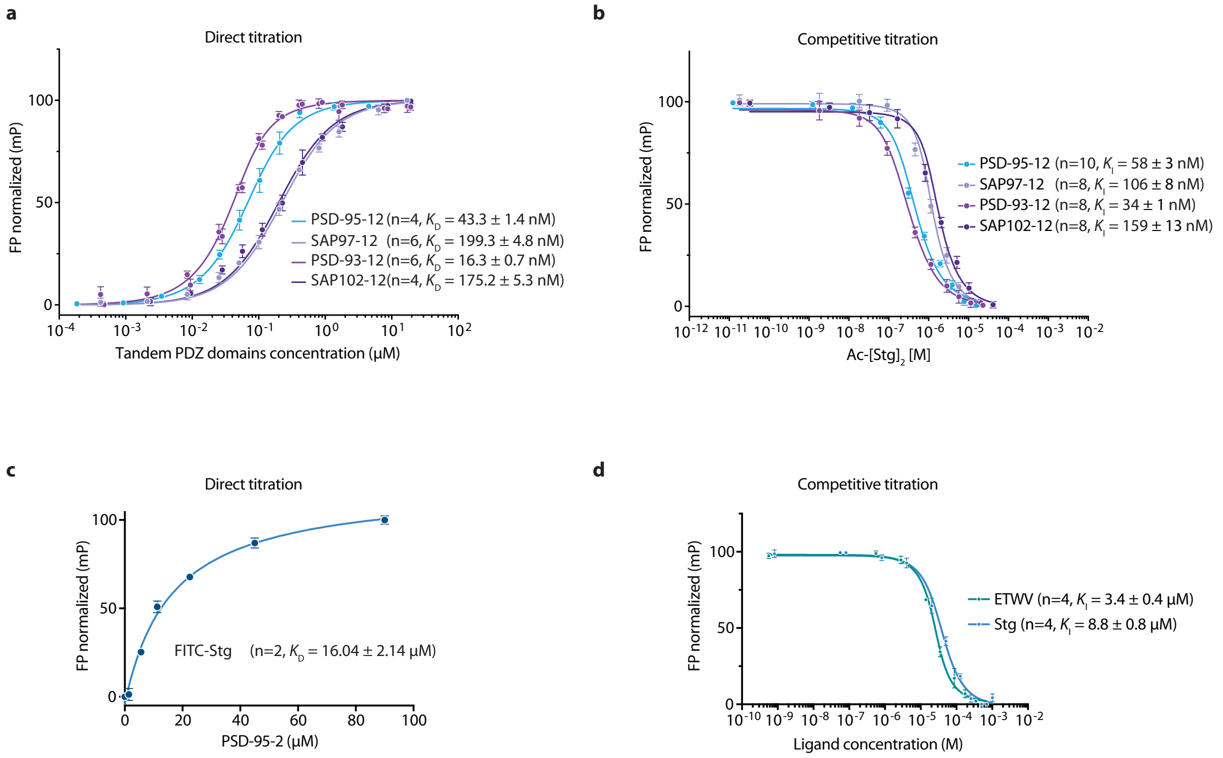

Supplementary Figure 28 | Fluorescence polarization titrations of di- and monovalent ligands against PDZ domains. **(a)** and **(b)** Divalent stargazin-derived ligand binding to tandem PDZ domains. **(a)** Direct titrations of FITC-derived stargazin divalent ligand (50 nM) against DLG tandem PDZ domains. Each data point represents the average of n independent measurements ± s.d. The dissociation constants obtained by fitting are reported with the calculated s.e.m. (**b**) Competitive titrations with non-fluorescent (acetylated) stargazin divalent ligand against the complexes between FITC-derived stargazin divalent ligand and the various tandem PDZ domains. Each data point represents the average of n independent measurements ± s.d. The inhibition constants obtained by fitting are reported with the calculated s.e.m. **(c)** and **(d)** Monovalent stargazin-derived ligand binding to PSD-95 second PDZ domain. **(c)** Direct titration of FITC-derived stargazin divalent ligand (50 nM) against PSD-95 second PDZ domain. Each data point represents the average of n independent measurements ± s.d. The dissociation constants obtained by fitting are reported with the calculated s.e.m. (**d**) Competitive titrations with non-fluorescent stargazin or ETWV monovalent ligand against the complex between FITC-derived stargazin monovalent ligand and PSD-95 second PDZ domain. Each data point represents the average of n independent measurements ± s.d. The inhibition constants obtained by fitting are reported with the calculated s.e.m. Source data are provided as Source Data file.

a

Xph20-ETWV sequence:

MASHHHHHHHHHHENLYFQSGSGSSVSSVPTKLEVVAATPTSLLISWDAVAKNVKVGYYRITYGETGG
NSPVQEFTVPGSSSTATISGLKPGVDYTITVYANGVLSKMVLPISINYRTLEGGSGGSGGSGGSGGSG
GSGGSGGSGGSGGSGGSGTETWV

b

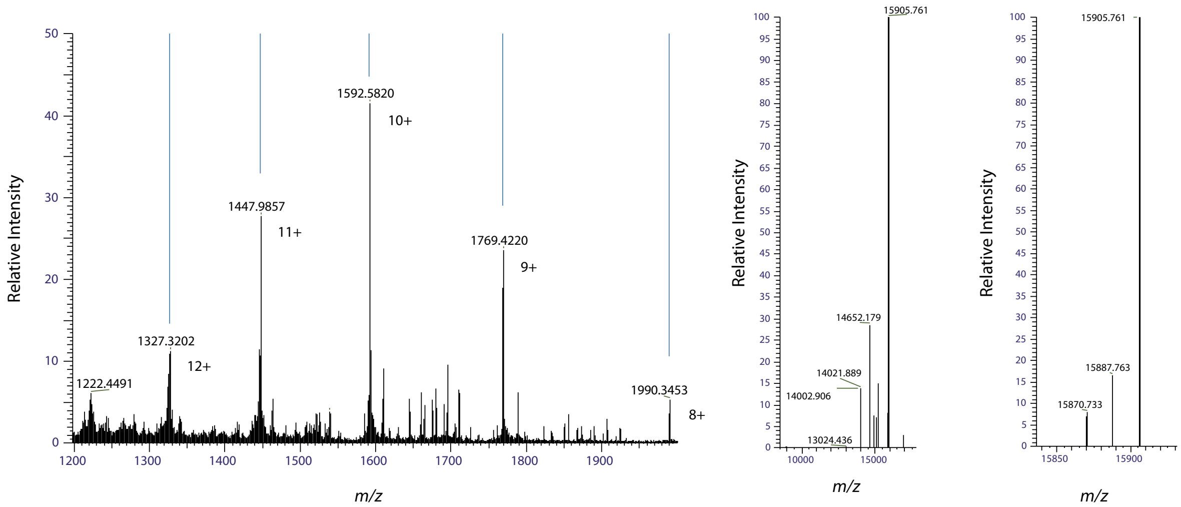

Supplementary Figure 29 | Mass spectroscopy analysis of Xph20-ETWV by LC-MS. **(a)** Primary sequence of Xph20-ETWV (purple letters: His_{10} tag; blue letters: Xph20; green letters: linker; red letters: ETWV PDZ domain-binding motif). **(b)** Raw (left) and deconvoluted (right) spectra.

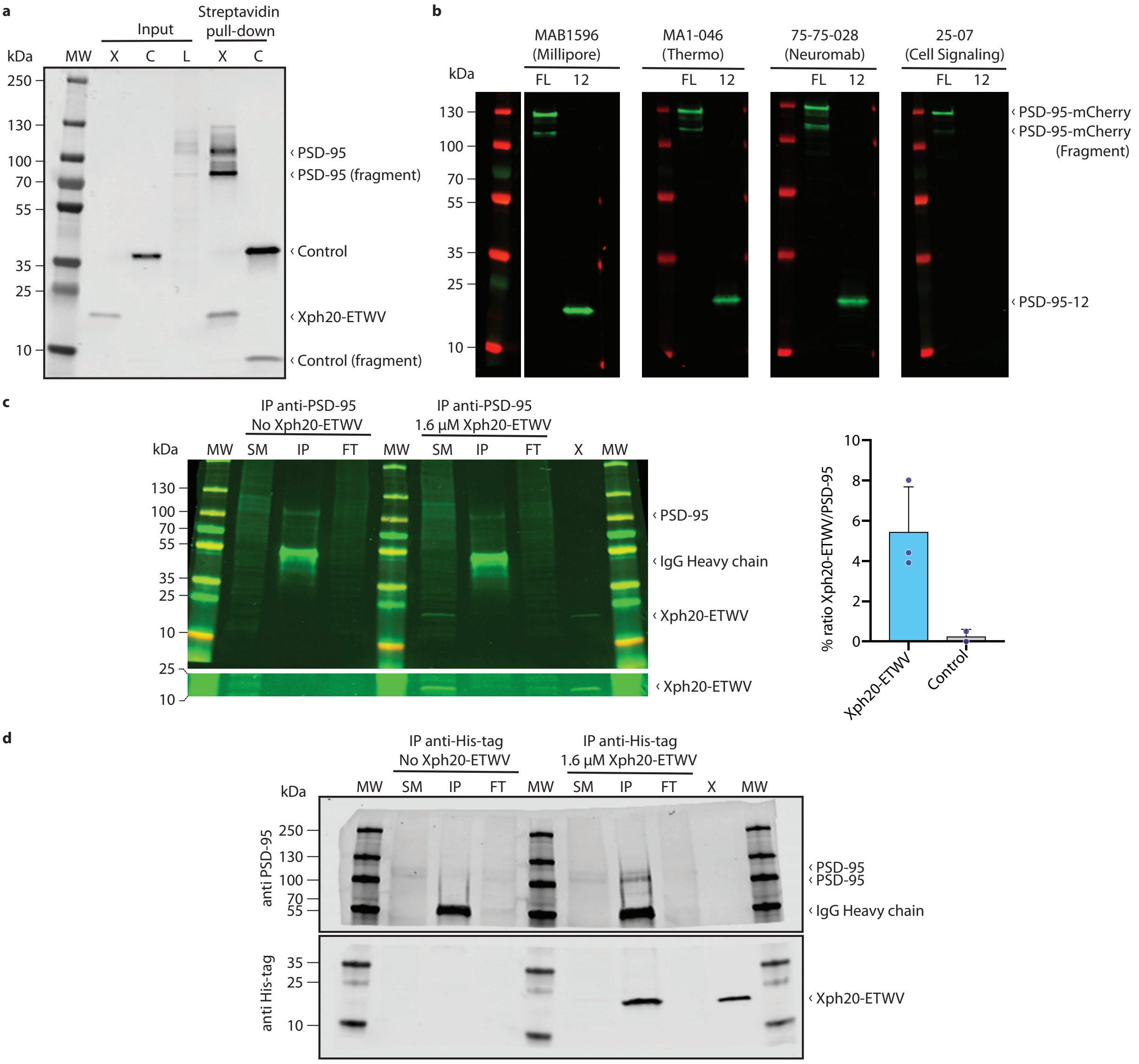

Supplementary Figure 30 | (a) Western blot analysis (uncropped gel from Fig 7a) with anti-PSD-95 (Millipore-Merck cat# MAB1596) and anti-His-tag (Sigma-Aldrich cat# H1029) of pull-down material from an adult rat brain lysate incubated with biotinylated Xph20-ETWV or control protein. X stands for Xph20-ETVW, C for control (non-binding fluorescent protein, mScarlet-i) and L for lysate. **(b)** Western blot analysis of various anti-PSD-95 antibodies against recombinant full-length PSD-95 (FL, PSD-95-mCherry) and tandem PDZ domains 1 and 2 (12). Most antibodies present epitopes in the tandem region with potential overlap with Xph20 epitopes. **(c)** and **(d)** Immunoprecipitation and western blot analysis of culture hippocampal neurons incubated with or without TAT-Xph20-ETWV. SM stands for starting material (lysate prior to IP), IP for the immunoprecipitated material, FT for the IP flow-through, and X for TAT-Xph20-ETWV. **(c)** Representative anti-PSD-95 IP (Cell Signaling cat# 2507) with blotting performed with anti-mouse Histidine tag (Sigma-Aldrich cat# H1029) and anti-mouse PSD-95 (Millipore-Merck cat# MAB1596). The 25-10 kDa region is reproduced at higher brightness and contrast. Left densitometric analysis of the area corresponding to Xph20-ETWV normalized by the area intensity of the corresponding PSD-95 band (mean + S.D., n=3). **(d)** Representative anti-His-tag IP (Abcam cat# ab18184) with blotting performed with anti-mouse Histidine tag (Sigma-Aldrich cat# H1029) and anti-mouse PSD-95 (Millipore-Merck cat# MAB1596) antibodies (uncropped gel from Fig 7b).

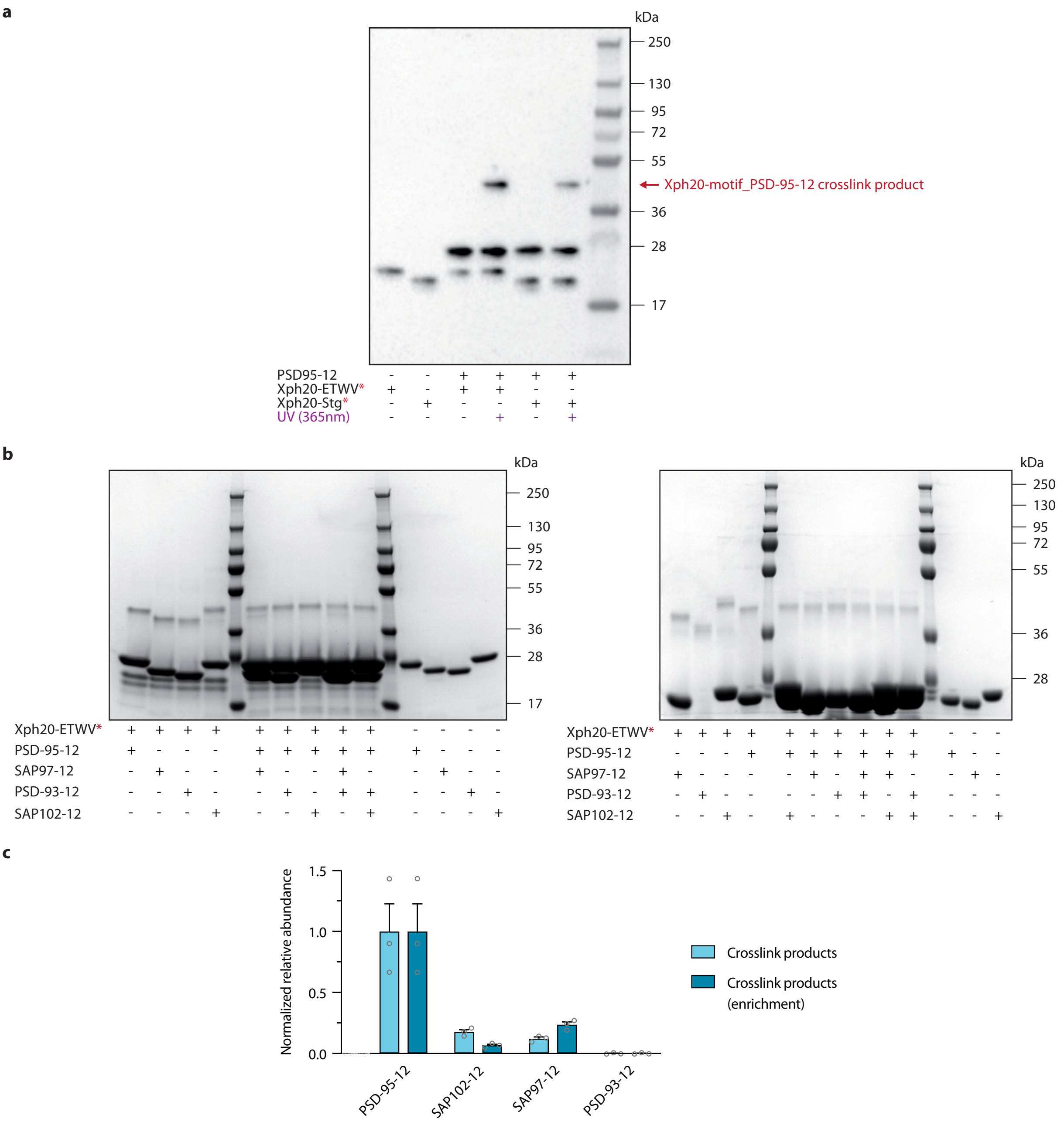

Supplementary Figure 31 | Analysis of photocrosslink assays. **(a)** Western Blot analysis (HisProbe HRP) of Xph20-ETWV* or Xph20-Stg* with PSD-95-12 (full blot from Fig 8b). In both cases, a unique band around 40 kDa appears after irradiation at 365 nm. **(b)** Colloidal blue-stained SDS-PAGE of the assays of Xph20-ETWV* with the indicated tandem PDZ domains (each used in a >3-fold excess). Left: representative gel with bands for all proteins visible; Right: representative gel with focus on the 55-36 kDa region. While individually each tandem PDZ domains can photocrosslink with Xph20-ETWV*, in the case of mixtures of tandem, PSD-95-12 is the major photocrosslink product observed indicating a strong selectivity. **(c)** Relative abundance of crosslink species determined by MS/MS analysis of the reaction of Xph20-ETWV* with a 3-fold excess of each of the 4 DLGs. Abundance was determined by considering only peptides fragments unique to each specie and the results are provided either as a direct comparison of the crosslink products or corrected for the measured composition of the non-crosslinked DLG mix (enrichment and correction for potential detection bias). The bars represent the mean of three independent analysis ± s.d. Source data are provided as Source Data file.

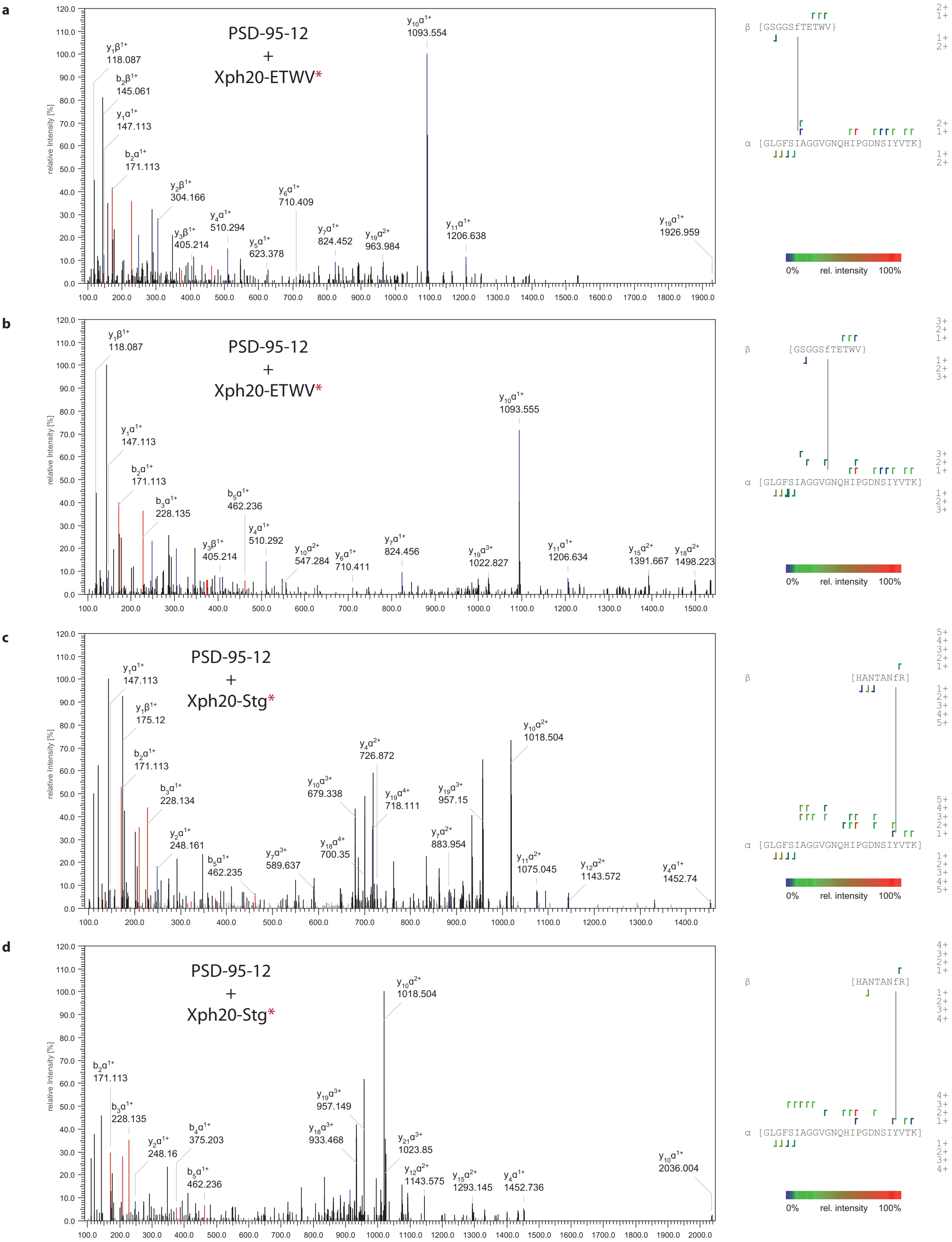

Supplementary Figure 32 | Mapping of crosslinks between pAzF-containing competitors and their targeted tandem PDZ domains. Bands corresponding to the crosslink product of Xph20-ETWV* or Xph20-Stg* with PSD-95-12 were excised, trypsin-digested and submitted to LC/MS^2. The results were analyzed with StavroX. Shown here are examples for the MS analysis of Xph20-ETWV* **(a)** and **(b)** (first and second analyzed fragments respectively from Table S4) and of Xph20-Stg* **(c)** and **(d)** (first and second analyzed fragments respectively from Table S5). "f" stands for *p*-azidophenylalanine.

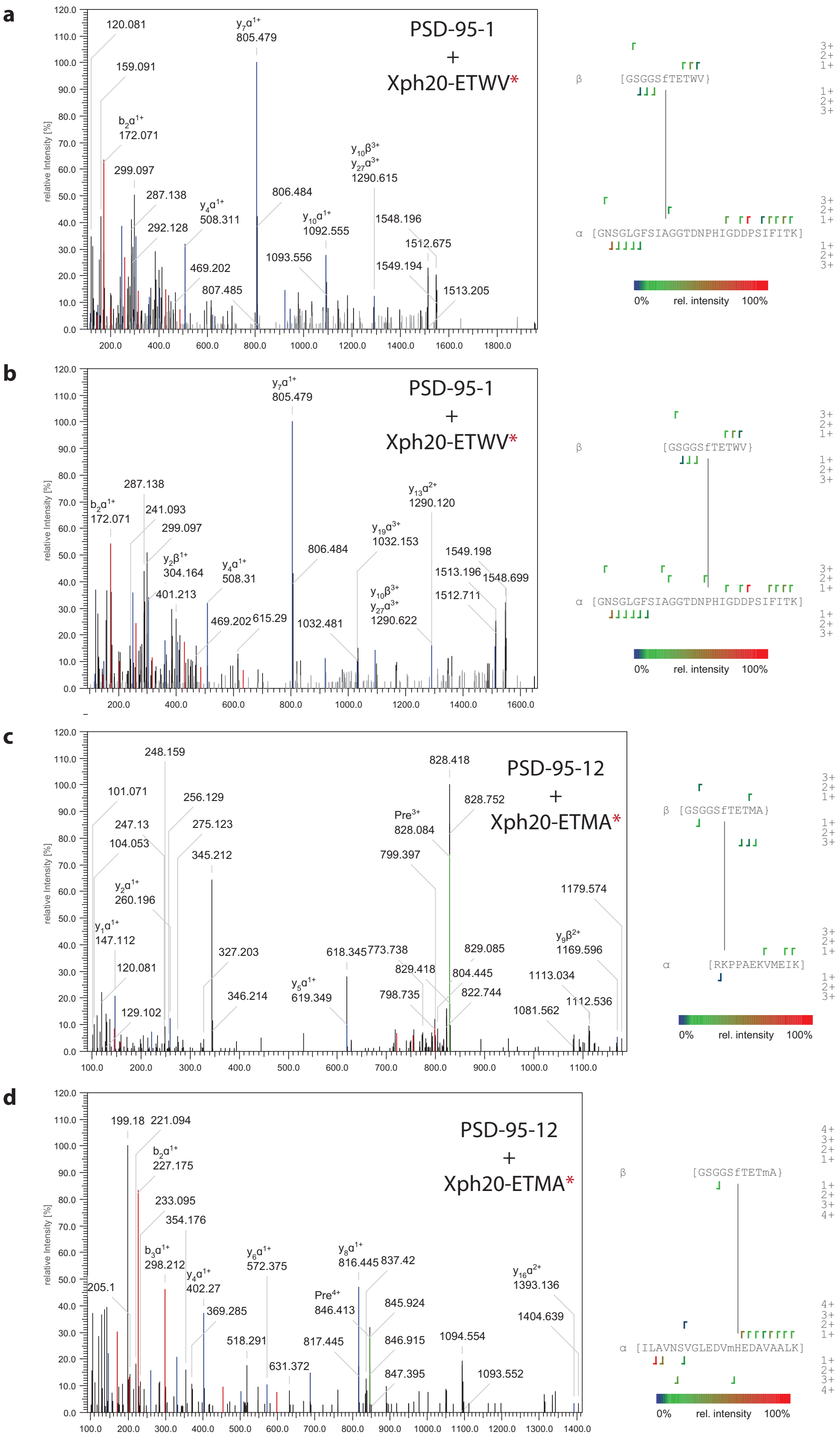

Supplementary Figure 33 | Mapping of crosslinks between pAzF-containing competitors and their targeted tandem PDZ domains. Bands corresponding to the crosslink product of Xph20-ETWV* or Xph20-ETMA* with PSD-95-1 and PSD-95-12 respectively were excised, trypsin-digested and submitted to LC/MS^2. The results were analyzed with StavroX. Shown here are examples for the MS analysis of Xph20-ETWV* **(a)** and **(b)** (first and second analyzed fragments respectively from Table S6) and of Xph20-ETMA* **(c)** and **(d)** (first and second analyzed fragments respectively from Table S7). "f" stands for *p*-azidophenylalanine.

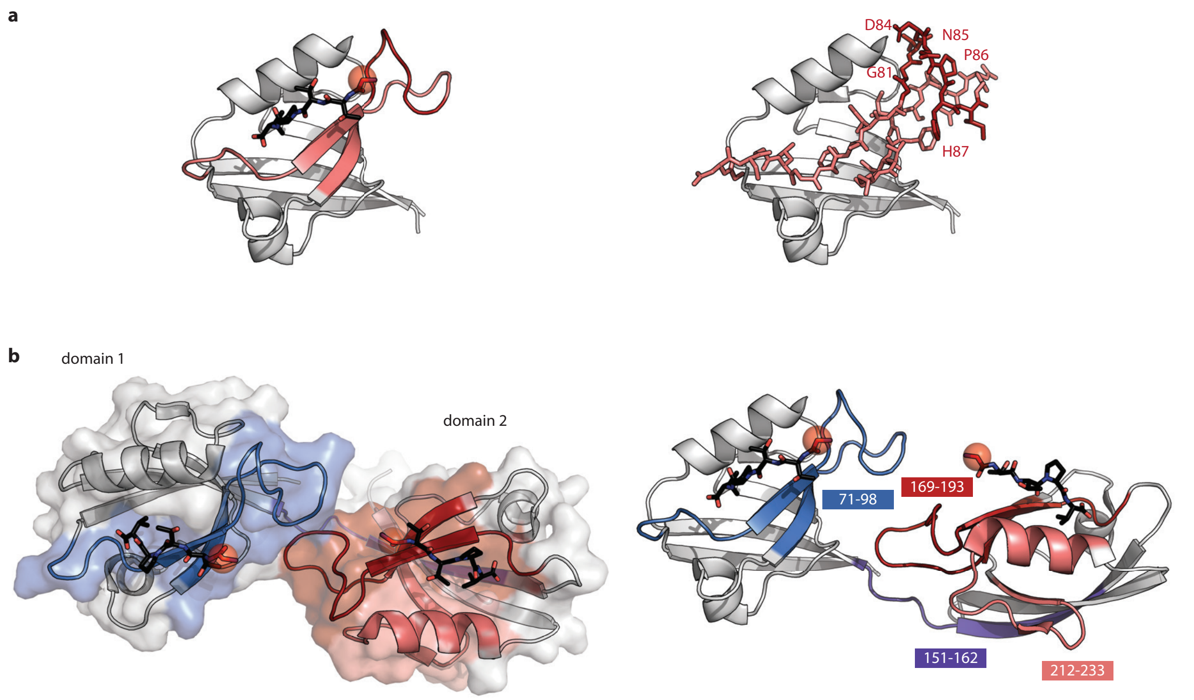

Supplementary Figure 34 | Position of PSD-95 photocrosslinked fragments with Xph20-ETWV* and -ETMA* identified by LC-MS/MS. PSD-95 domains1 and 2 (PDB ID 3GSL) with ligands modelled in and occupying the binding grooves (xTTPV, black sticks). The red spheres represent pAzF position in the binding motifs (x position). **(a)** Mapping of the photocrosslinked product for Xph20-ETWV* with PSD-95 PDZ domain 1. The salmon and red carton and sticks represent the identified fragment with the red highlighting the most likely candidate residues. **(b)** Mapping of the photocrosslinked product for Xph20-ETMA* with PSD-95 PDZ domains 1 and 2. The four main fragments identified by tandem MS are represented in blue (residues 71-98), purple (151-162), red (169-193) and salmon (212-233).

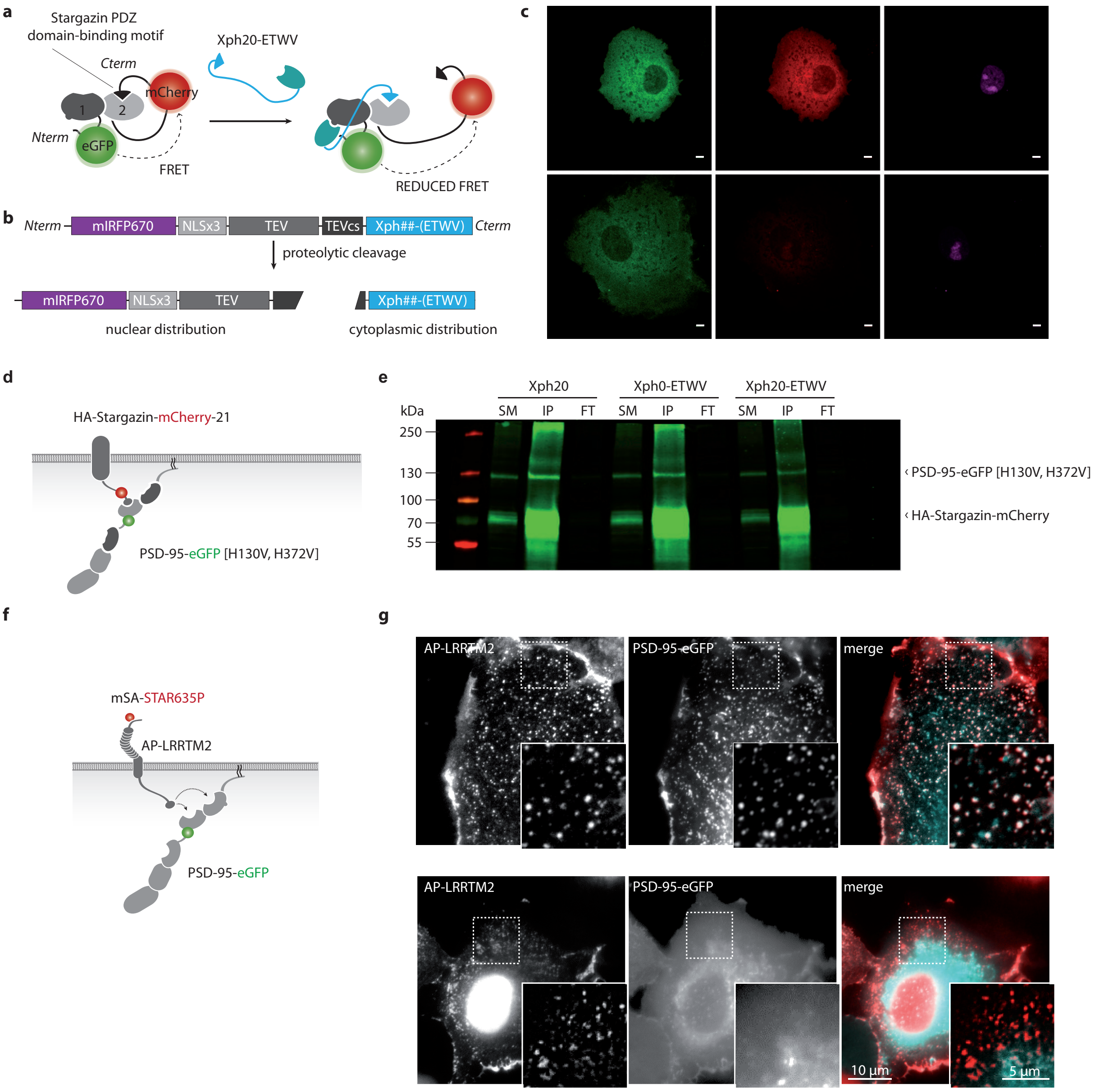

Supplementary Figure 35 | Competition in cellular environment **(a)** Design and principle of PSD-95 PDZ domain 2 intramolecular FRET reporter. Presence of PDZ domain 1 in a mutated form (H130V) is key to allow Xph20 binding while impairing direct interaction of the reporter PDZ domain-binding motif. **(b)** General design for the competitor expression. After gene synthesis, the TEV protease cuts the TEV cleavage site (TEVcs) resulting in equimolar infrared mIRFP670-Nuc-TEV (targeted to the nucleus) and competitor (remaining in the cytoplasm). This allows for a precise correlation between the expression levels of mIRFP670 and the competitor or control. Of note, initial attempts in COS-7 cells with P2A and T2A self-cleaving peptides failed to provide us with quantitative cleavage. **(c)** Representative images of FRET reporter (green and red) and competitor (magenta, Xph20-ETWV) expressing COS-7 cells. Scale bar represents 5 µm. **(d)** Scheme for the co-immunoprecipitation experiment. As stargazin can bind efficiently all three PSD-95 PDZ domains, a double mutant (H130V, H372V) was used to impair the binding properties of PDZ domains 1 and 3. **(e)** Representative Western blot for the co-immunoprecipitation experiments (fluorescence detection, exposure optimized for PSD-95 quantification). **(f)** Scheme for the detection of LRRTM2 and PSD-95 detection in the clustering assay. AP-LRRTM2 is detected using monomeric streptavidin (mSA) conjugated to

STAR635P when co-expressed with BirA-ER. **(g)** Representative images of COS cells expressing AP-LRRTM2, BirA-ER, PSD-95-GFP without (top) or with (bottom) Xph20-ETWV. In the absence of Xph20-ETWV, PSD-95 forms clusters colocalized with LRRTM2 (top). In the presence of Xph20-ETWV, PSD-95 displays a diffuse staining (bottom).

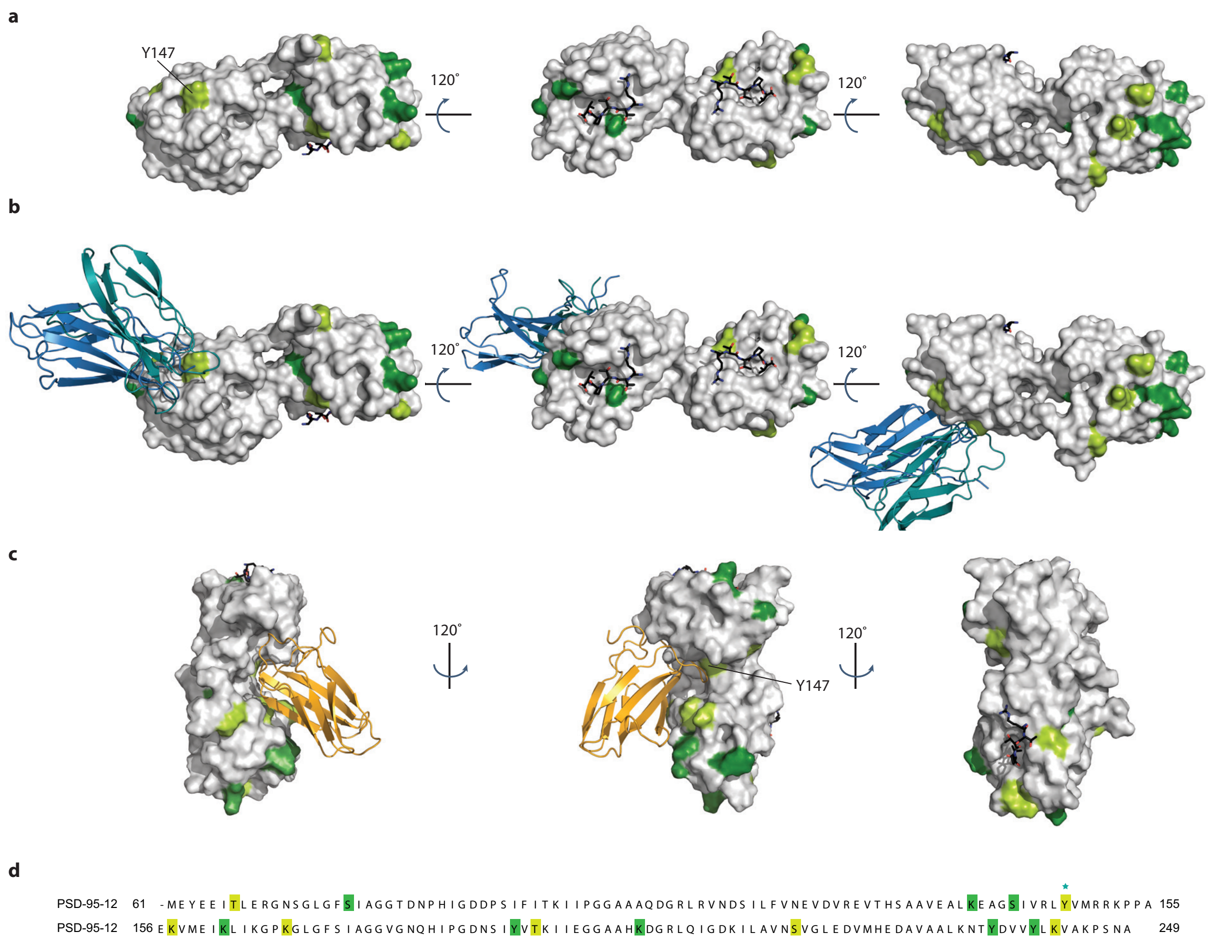

Supplementary Figure 36 | (a) Post-translational modifications (PTMs) and Xph15/18/20 epitopes. **(a)** Mapping of reported PTMs (in green) for PSD-95 on domain 1 and 2 structure (adapted from 3GSL with models of the ligands in black stick located in the two binding grooves). Dark and light green correspond to PTMs that have been reported by more than five different sources or by less than five sources respectively. List of PTMs for PSD-95 were obtained from the PhosphositePlus database (www.phosphosite.org). **(b)** Model of Xph15 (blue) and Xph20 (teal) bound to PSD-95 domain 1 and 2. Same orientations as **a**. for comparison. **(c)** Model of Xph18 (orange) bound to PSD-95 domain 1 and 2. Domain 1 on top. **(d)** PTMs positions on the primary sequence of PSD-95 domains 1 (left) and 2 (right) with same colour code as in **a**. The asterisks correspond to a potential conflicting position (the Y147 phosphorylation site has only been observed once in a proteomic screen with no indication of its biological relevance).

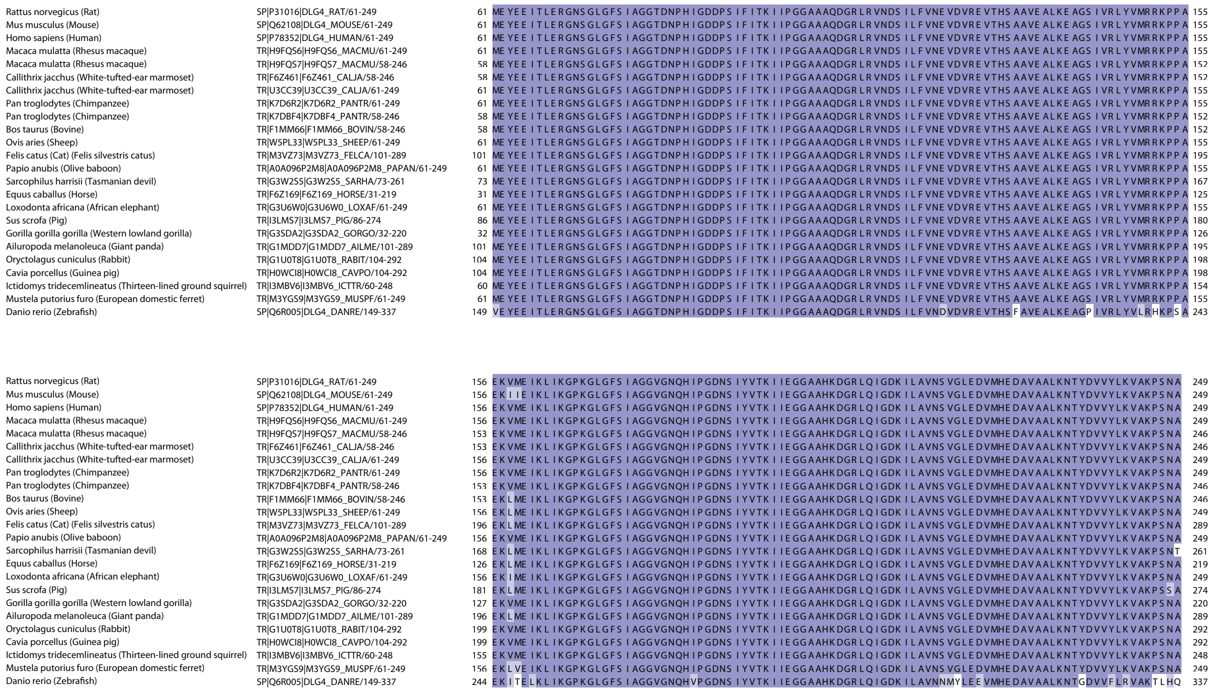

Supplementary Figure 37 | Sequence alignment of PSD-95 domains 1 and 2 across species (obtained from the Uniprot database).

Supplementary Table 1 | Binding and kinetic constants measured by SPR for binding of competitors to tandems of the first two PDZ domains of the PSD-95 family. [a] Dissociation constants determined by kinetics analysis of single cycle kinetics experiments (average ± s.d., n ≥ 2 replicates). [b] Dissociation constants determined by steady state analysis of single cycle kinetics experiments (fitted value ± standard error, n ≥ 2). [c] n.d. indicates that the values were not determined.

Ligand	PSD-95-12			SAP97-12	PSD-93-12	SAP102-12
	k_{on} (1/Ms)	k_{off} (1/s)	K_D (M)[a]	K_D (M)[b]	K_D (M)[b]	K_D (M)[b]
Xph15-ETWV	$3.7 \pm 0.5 \times 10^{6}$	$9.1 \pm 0.2 \times 10^{-4}$	$2.5 \pm 0.4 \times 10^{-10}$	$2.2 \pm 0.4 \times 10^{-6}$	$8.4 \pm 0.8 \times 10^{-6}$	$9.8 \pm 0.8 \times 10^{-6}$
Xph18-ETWV	$2.3 \pm 1.0 \times 10^{6}$	$3.4 \pm 2.0 \times 10^{-4}$	$2.0 \pm 1.7 \times 10^{-10}$	$1.6 \pm 0.3 \times 10^{-6}$	$3.2 \pm 0.4 \times 10^{-6}$	$4.2 \pm 0.5 \times 10^{-6}$
Xph20-ETWV	$8.7 \pm 8.2 \times 10^{6}$	$5.2 \pm 2.0 \times 10^{-4}$	$8.9 \pm 6.2 \times 10^{-11}$	$1.5 \pm 0.2 \times 10^{-6}$	$7.5 \pm 0.5 \times 10^{-6}$	$9.9 \pm 0.5 \times 10^{-6}$
Xph18-Stg	$6.1 \pm 5.7 \times 10^{7}$	$1.5 \pm 0.8 \times 10^{-3}$	$3.4 \pm 2.0 \times 10^{-11}$	n.d.[c]	n.d.[c]	n.d.[c]
Xph20-Stg	$1.8 \pm 0.7 \times 10^{7}$	$9.0 \pm 0.4 \times 10^{-4}$	$5.4 \pm 2.4 \times 10^{-11}$	n.d.[c]	n.d.[c]	n.d.[c]

Supplementary Table 2 | Inhibition constants and corresponding 95% confidence interval from competitive titrations of Xph20-ETWV against a complex of tandem PDZ domains and a divalent ligand (Fig 6c).

Xph#-ETWV	Xph0	Xph20	Xph20	Xph20	Xph20
PDZ domains	PSD-95-12	SAP102-12	PSD-93-12	SAP97-12	PSD-95-12
K_I (nM)	821.0	1443	261.2	900.8	36.46
95 % CI (nM)	624.9 to 1088	990.6 to 2129	192.8 to 355.8	633.6 to 1293	29.03 to 45.70

Supplementary Table 3 | Analysis of the main species observed by mass spectrometry for Xph20-ETWV. The analysis of potential unspecific cleavages was performed using FindPept (ExPaSy, https://web.expasy.org/findpept/). Grey letters correspond to digested residues, red letters correspond to the PDZ domain-binding motif.

Monoisotopic Mass	Relative Abundance	Δmass	Predicted mass	Δmass (pred.-obs.)	Predicted peptide	position
15905.761	100	0	15907.683	1.922	(M)ASHHHHHHHHHHENLYFQSG S...LEG GSGGSGGSGGSGGSGGSGGS GGSGGSGGSGGSGGSGTETW V	2-162
14652.179	28.54	-1253.582	14651.141	-1.038	(H)HHHHHHHHHENLYFQSGS...LEGGSG GSGGSGGSGGSGGSGGSGGS GGSGGSGG(S)	5-152
			14653.143	0.963	(H)HHENLYFQSGS...LEGGSGGSGGSGG SGGSGGSGGSGGSGGSGGSG GSGGSGTETWV	12-162
15887.763	16.7	-17.998				
15241.429	15.05	-664.333	15239.369	-2.06	(H)HHHHHHHENLYFQSGS...LEGGSGGS GGSGGSGGSGGSGGSGGSGG SGGSGGSGGSGTETW(V)	7-161
14021.889	13.78	-1883.872	14022.855	0.966	(L)YFQSGS...LEGGSGGSGGSGGSGGSG GSGGSGGSGGSGGSGGSGGS GTETWV	17-162

Supplementary Table 4 | Mapping of the crosslink position in PSD-95-12 with Xph20-ETWV* using tandem-MS and analysis with StavroX. MS spectra of grey shaded entries are shown in Figure S31a and b. Red letters indicate highest probable crosslink candidates. "f" = *p*-azidophenylalanine.

Peptide β	Score	m/z	Charge	M+H+	Calculated Mass	Deviation in ppm	Peptide α	From	To	best linkage position peptide α
[GSGGSfTETWV}	119	910.950	4	3640.777	3640.767	2.84	[GLGFSIAGGVGNQHIPGDNSIYVTK]	127	151	I6
	117	910.952	4	3640.787	3640.767	5.65	[GLGFSIAGGVGNQHIPGDNSIYVTK]	127	151	G11
	107	910.949	4	3640.773	3640.767	1.77	[GLGFSIAGGVGNQHIPGDNSIYVTK]	127	151	I6
	106	910.950	4	3640.777	3640.767	2.78	[GLGFSIAGGVGNQHIPGDNSIYVTK]	127	151	G11
	92	1214.266	3	3640.785	3640.767	4.91	[GLGFSIAGGVGNQHIPGDNSIYVTK]	127	151	I6
	88	728.958	5	3640.762	3640.767	-1.24	[GLGFSIAGGVGNQHIPGDNSIYVTK]	127	151	F4
	85	1214.257	3	3640.756	3640.767	-2.84	[GLGFSIAGGVGNQHIPGDNSIYVTK]	127	151	G1
	84	910.950	4	3640.780	3640.767	3.58	[GLGFSIAGGVGNQHIPGDNSIYVTK]	127	151	I6
	82	1214.262	3	3640.772	3640.767	1.49	[GLGFSIAGGVGNQHIPGDNSIYVTK]	127	151	F4

Supplementary Table 5 | Mapping of the crosslink position in PSD-95-12 with Xph20-Stg* using tandem-MS. MS spectra of grey shaded entries are shown in Figure S31c and d. Red letters indicate highest probable crosslink candidates. Grey letters indicate crosslinks with the N-terminus of PSD-95-12, which are not specifically driven by interaction of the PDZ domain-binding motif. "m" = oxidized methionine, "f" = *p*-azidophenylalanine.

Peptide β	Score	m/z	Charge	M+H⁺	Calculated Mass	Deviation in ppm	Peptide α	From	To	best linkage position peptide α
[HANTANfR]	125	689.552	5	3443.732	3443.720	3.43	[GLGFSIAGGVGNQHIPGDNSIYVTK]	127	151	V23
	114	861.687	4	3443.727	3443.720	1.92	[GLGFSIAGGVGNQHIPGDNSIYVTK]	127	151	V23
	111	689.555	5	3443.745	3443.720	7.06	[GLGFSIAGGVGNQHIPGDNSIYVTK]	127	151	V23
	110	647.148	7	4523.994	4524.003	-2.08	{mHHHHHHHHENLYFQSGSMEYEEITLER]	0	28	H4
	106	752.170	6	4507.985	4508.008	-5.06	{MHHHHHHHHENLYFQSGSMEYEEITLER]	0	28	M1
	104	861.688	4	3443.729	3443.720	2.64	[GLGFSIAGGVGNQHIPGDNSIYVTK]	127	151	H14
	104	689.550	5	3443.721	3443.720	0.33	[GLGFSIAGGVGNQHIPGDNSIYVTK]	127	151	I21
	100	752.173	6	4508.003	4508.008	-1.08	{MHHHHHHHHENLYFQSGSMEYEEITLER]	0	28	M1
	100	647.145	7	4523.972	4524.003	-6.89	{mHHHHHHHHENLYFQSGSMEYEEITLER]	0	28	H2
	96	647.148	7	4523.994	4524.003	-2.08	{MHHHHHHHHENLYFQSGSmEYEEITLER]	0	28	M1
	94	754.838	6	4523.992	4524.003	-2.36	{mHHHHHHHHENLYFQSGSMEYEEITLER]	0	28	H4
	93	574.794	6	3443.728	3443.720	2.35	[GLGFSIAGGVGNQHIPGDNSIYVTK]	127	151	N19
	93	754.838	6	4523.992	4524.003	-2.36	{MHHHHHHHHENLYFQSGSmEYEEITLER]	0	28	H3
	93	647.145	7	4523.972	4524.003	-6.89	{MHHHHHHHHENLYFQSGSmEYEEITLER]	0	28	M1
	93	754.841	6	4524.013	4524.003	2.10	{mHHHHHHHHENLYFQSGSMEYEEITLER]	0	28	H4
	89	689.550	5	3443.721	3443.720	0.15	[GLGFSIAGGVGNQHIPGDNSIYVTK]	127	151	P16
	88	861.688	4	3443.729	3443.720	2.64	[GLGFSIAGGVGNQHIPGDNSIYVTK]	127	151	A7
	86	754.841	6	4524.013	4524.003	2.10	{MHHHHHHHHENLYFQSGSmEYEEITLER]	0	28	M1
	85	574.795	6	3443.732	3443.720	3.41	[GLGFSIAGGVGNQHIPGDNSIYVTK]	127	151	Y22

Supplementary Table 6 | Mapping of the crosslink position in PSD-95-1 with Xph20-ETWV* using tandem-MS. MS spectra of the two entries are shown in Figure S32a and b. Red letters indicate highest probable crosslink candidates. "f" = *p*-azidophenylalanine.

Peptide β	Score	m/z	Charge	M+H+	Calculated Mass	Deviation in ppm	Peptide α	From	To	best linkage position peptide α
[GSGGSfTETWV}	87	982.469	4	3926.853	3926.847	1.69	[GNSGLGFSIAGGTDNPHIGDDPSIFITK]	29	56	F7
	82	982.468	4	3926.850	3926.847	0.77	[GNSGLGFSIAGGTDNPHIGDDPSIFITK]	29	56	P16

Supplementary Table 7 | Mapping of the crosslink position in PSD-95-12 with Xph20-ETMA* using tandem-MS. MS spectra of grey shaded entries are shown in Figure S32c and d. Grey letters indicate crosslinks with the N-terminus of PSD-95-12, which are not specifically driven by interaction of the PDZ domain-binding motif. "m" = oxidized methionine, "f" = *p*-azidophenylalanine.

Peptide β	Score	m/z	Charge	M+H+	Calculated Mass	Deviation in ppm	Peptide α	From	To	best linkage position peptide α
[GSGGSfTETMA}	99	828.086	3	2482.245	2482.243	0.83	[RKPPAEKVMEIK]	109	120	K7
[GSGGSfTETmA}	96	846.410	4	3382.617	3382.614	0.88	[ILAVNSVGLEDVmHEDAVAALK]	170	191	H14
[GSGGSfTETMA}	77	838.409	4	3350.612	3350.624	-3.53	[ILAVNSVGLEDVMHEDAVAALK]	170	191	V12
[GSGGSfTETmA}	76	1191.895	3	3573.671	3573.691	-5.86	[GLGFSIAGGVGNQHIPGDNSIYVTK]	127	151	I6
[GSGGSfTETMA}	76	961.697	4	3843.767	3843.777	-2.51	[GNSGLGFSIAGGTDNPHIGDDPSIFITK]	29	56	P16
[GSGGSfTETmA}	75	1191.899	3	3573.683	3573.691	-2.28	[GLGFSIAGGVGNQHIPGDNSIYVTK]	127	151	G8
[GSGGSfTETMA}	75	890.181	4	3557.701	3557.697	1.20	[GLGFSIAGGVGNQHIPGDNSIYVTK]	127	151	G11
[GSGGSfTETmA}	74	894.178	4	3573.690	3573.691	-0.45	[GLGFSIAGGVGNQHIPGDNSIYVTK]	127	151	A7
[GSGGSfTETmA}	73	842.408	4	3366.609	3366.619	-2.94	[ILAVNSVGLEDVMHEDAVAALK]	170	191	H14
[GSGGSfTETMA}	73	890.173	4	3557.671	3557.697	-7.23	[GLGFSIAGGVGNQHIPGDNSIYVTK]	127	151	I6
[GSGGSfTETmA}	73	965.705	4	3859.798	3859.772	6.79	[GNSGLGFSIAGGTDNPHIGDDPSIFITK]	29	56	G11
[GSGGSfTETMA}	72	961.699	4	3843.774	3843.777	-0.80	[GNSGLGFSIAGGTDNPHIGDDPSIFITK]	29	56	L5
[GSGGSfTETMA}	71	771.169	6	4621.977	4621.984	-1.69	{MHHHHHHHHENLYFQSGSMEYEEITLER]	0	28	E10
[GSGGSfTETMA}	70	771.170	6	4621.986	4621.984	0.29	{MHHHHHHHHENLYFQSGSMEYEEITLER]	0	28	E10
[GSGGSfTETmA}	70	1191.901	3	3573.689	3573.691	-0.74	[GLGFSIAGGVGNQHIPGDNSIYVTK]	127	151	I6
[GSGGSfTETmA}	70	1191.901	3	3573.689	3573.691	-0.63	[GLGFSIAGGVGNQHIPGDNSIYVTK]	127	151	F4
[GSGGSfTETmA}	69	1191.894	3	3573.667	3573.691	-6.88	[GLGFSIAGGVGNQHIPGDNSIYVTK]	127	151	I6
[GSGGSfTETmA}	69	965.697	4	3859.765	3859.772	-1.68	[GNSGLGFSIAGGTDNPHIGDDPSIFITK]	29	56	G11
[GSGGSfTETMA}	69	838.409	4	3350.614	3350.624	-3.17	[ILAVNSVGLEDVMHEDAVAALK]	170	191	G8
[GSGGSfTETmA}	67	894.177	4	3573.688	3573.691	-1.00	[GLGFSIAGGVGNQHIPGDNSIYVTK]	127	151	G8
[GSGGSfTETMA}	66	771.167	6	4621.965	4621.984	-4.15	{MHHHHHHHHENLYFQSGSMEYEEITLER]	0	28	E10
[GSGGSfTETMA}	66	928.401	5	4637.978	4637.979	-0.27	{mHHHHHHHHENLYFQSGSMEYEEITLER]	0	28	H2
[GSGGSfTETmA}	66	1191.893	3	3573.665	3573.691	-7.30	[GLGFSIAGGVGNQHIPGDNSIYVTK]	127	151	I6
[GSGGSfTETmA}	66	965.698	4	3859.769	3859.772	-0.68	[GNSGLGFSIAGGTDNPHIGDDPSIFITK]	29	56	G11
[GSGGSfTETmA}	66	965.696	4	3859.761	3859.772	-2.70	[GNSGLGFSIAGGTDNPHIGDDPSIFITK]	29	56	N15
[GSGGSfTETMA}	66	925.203	5	4621.988	4621.984	0.86	{MHHHHHHHHENLYFQSGSMEYEEITLER]	0	28	E10
[GSGGSfTETMA}	65	724.337	3	2170.996	2170.996	-0.14	[NTYDVVYLK]	192	200	V6
[GSGGSfTETMA}	65	771.168	6	4621.970	4621.984	-3.03	{MHHHHHHHHENLYFQSGSMEYEEITLER]	0	28	E10
[GSGGSfTETMA}	65	842.409	4	3366.616	3366.619	-0.92	[ILAVNSVGLEDVmHEDAVAALK]	170	191	H14
[GSGGSfTETmA}	65	931.594	5	4653.942	4653.974	-6.90	{MHHHHHHHHENLYFQSGSmEYEEITLER]	0	28	M1

Supplementary Table 8 | Effective concentration (C_{eff}) determination. The affinities of the two binding motifs (ETWV and Stg) were determined by a competitive fluorescence polarization assay (Supplementary Fig 28). $C_{eff} = (K_D{}^{motif} \times K_D{}^{Xph}) / K_D{}^{fusion} \approx (K_I{}^{motif} \times K_D{}^{Xph}) / K_D{}^{fusion}$

	$K_D{}^{fusion}$ (M)	$K_I{}^{motif}$ (M)	$K_D{}^{Xph}$ (M)	C_{eff} (mM)
Xph15-ETWV	2.50×10^{-10}	3.36×10^{-6}	4.30×10^{-6}	57.8
Xph18-ETWV	2.00×10^{-10}	3.36×10^{-6}	2.60×10^{-6}	43.7
Xph20-ETWV	8.90×10^{-11}	3.36×10^{-6}	3.30×10^{-7}	12.5
Xph18-Stg	3.40×10^{-11}	8.60×10^{-6}	2.60×10^{-6}	658
Xph20-Stg	5.40×10^{-11}	8.60×10^{-6}	3.30×10^{-7}	52.6

Supplementary Table 9 | List of plasmids used in this work. TEV_{cs} = TEV protease cleavage site; $Thrombin_{cs}$ = Thrombin protease cleavage site; c = commercial source; black dot = this study.

Entry	Plasmid name	Plasmid backbone	Origin of replication	Promoter	Gene	Purpose	Tag(s)	Figure(s)	Source
p01	pSEX81	pSEX81	ColE1/M13 f1	LacZ	Ø	Molecular biology	—	—	c
p02	pSEX84-Xph-0	pSEX84	ColE1/M13 f1	LacZ	Xph0	Phage Display	—	2a, 2b, S3	•
p03	pSEX84-Xph-15	pSEX84	ColE1/M13 f1	LacZ	Xph15	Phage ELISA	—	2b, 2c, S3	•
p04	pSEX84-Xph-16	pSEX84	ColE1/M13 f1	LacZ	Xph16	Phage ELISA	—	2b, 2c, S3	•
p05	pSEX84-Xph-17	pSEX84	ColE1/M13 f1	LacZ	Xph17	Phage ELISA	—	2b, 2c, S3	•
p06	pSEX84-Xph-18	pSEX84	ColE1/M13 f1	LacZ	Xph18	Phage ELISA	—	2b, 2c, S3	•
p07	pSEX84-Xph-19	pSEX84	ColE1/M13 f1	LacZ	Xph19	Phage ELISA	—	2b, 2c, S3	•
p08	pSEX84-Xph-20	pSEX84	ColE1/M13 f1	LacZ	Xph20	Phage ELISA	—	2b, 2c, S3	•
p09	pSEX84-Xph-21	pSEX84	ColE1/M13 f1	LacZ	Xph21	Phage ELISA	—	2b, 2c, S3	•
p10	pSEX84-Xph-22	pSEX84	ColE1/M13 f1	LacZ	Xph22	Phage ELISA	—	2c, S3	•
p11	pSEX84-Xph-23	pSEX84	ColE1/M13 f1	LacZ	Xph23	Phage ELISA	—	2c, S3	•
p12	pSEX84-Xph-24	pSEX84	ColE1/M13 f1	LacZ	Xph24	Phage ELISA	—	2b, 2c, S3	•
p13	pSEX84-Xph-25	pSEX84	ColE1/M13 f1	LacZ	Xph25	Phage ELISA	—	2b, 2c, S3	•
p14	pACYC-mCh-BirA	pACYC-duet-1	p15A	T7	BirA	Protein production	mCherry Nterminal	—	•
p15	pET-24a(+)	pET-24a(+)	pBR322	T7	Ø	Molecular biology	T7 tag; His_6	—	c
p16	pbIG-PSD-95-12	pbIG	pBR322	T7	PSD-95-12 [61-249]	Phage ELISA, SPR	AviTag; His_{10}; TEV_{cs}	2c, S3, S23, S25a	•
p17	pbIG-SAP97-12	pbIG	pBR322	T7	SAP97-12 [220-408]	Phage ELISA, SPR	AviTag; His_{10}; TEV_{cs}	2c, S3, S23, S25b	•
p18	pbIG-SAP102-12	pbIG	pBR322	T7	SAP102-12 [145-333]	Phage ELISA, SPR	AviTag; His_{10}; TEV_{cs}	2c, S3, S23	•
p19	pbIG-PSD-93-12	pbIG	pBR322	T7	PSD-93-12 [93-282]	Phage ELISA, SPR	AviTag; His_{10}; TEV_{cs}	2c, S3, S23	•
p20	pbIG-PSD-95-2	pbIG	pBR322	T7	PSD-95-2 [155-249]	Phage ELISA	AviTag; His_{10}; TEV_{cs}	S3	•
p21	pET-IG-Xph15	pET-IG	pBR322	T7	Xph15	Pull-Down, FPLC	His_{10}; TEV_{cs}	2d, S4, S6	•
p22	pET-IG-Xph16	pET-IG	pBR322	T7	Xph16	Pull-Down	His_{10}; TEV_{cs}	2d, S4	•
p23	pET-IG-Xph17	pET-IG	pBR322	T7	Xph17	Pull-Down	His_{10}; TEV_{cs}	2d, S4	•
p24	pET-IG-Xph18	pET-IG	pBR322	T7	Xph18	Pull-Down	His_{10}; TEV_{cs}	2d, S4	•
p25	pET-IG-Xph19	pET-IG	pBR322	T7	Xph19	Pull-Down	His_{10}; TEV_{cs}	2d, S4	•
p26	pET-IG-Xph20	pET-IG	pBR322	T7	Xph20	Pull-Down, FPLC	His_{10}; TEV_{cs}	2d, S4, S6	•
p27	pET-IG-Xph21	pET-IG	pBR322	T7	Xph21	Pull-Down	His_{10}; TEV_{cs}	2d, S4	•
p28	pET-IG-Xph24	pET-IG	pBR322	T7	Xph24	Pull-Down	His_{10}; TEV_{cs}	2d, S4	•
p29	pET-IG-Xph25	pET-IG	pBR322	T7	Xph25	Pull-Down	His_{10}; TEV_{cs}	2d, S4	•
p30	pGEX-PSD-95-12	pGEX-4T-2	pBR322	tac	PSD-95-12 [61-249]	Pull-Down	GST; TEV_{cs}; $Thrombin_{cs}$; Flag	2d, S4	1
p31	pGEX-SAP97-12	pGEX-4T-2	pBR322	tac	SAP97-12 [220-408]	Pull-Down	GST; TEV_{cs}; $Thrombin_{cs}$; Flag	2d, S4	1
p32	pGEX-SAP102-12	pGEX-4T-2	pBR322	tac	SAP102-12 [145-333]	Pull-Down	GST; TEV_{cs}; $Thrombin_{cs}$; Flag	2d, S4	1
p33	pGEX-PSD-93-12	pGEX-4T-2	pBR322	tac	PSD-93-12 [93-282]	Pull-Down	GST; TEV_{cs}; $Thrombin_{cs}$; Flag	2d, S4	•
p34	pEGFP-C1	pEGFP-C1	SV40	CMV	eGFP	FRET	eGFP	2f, S5b	c
p35	Stargazin mCherry [-21]	pcDNA3	SV40	CMV	Stargazin	FRET	mCherry at position -21	2f, S5b-c	2
p36	PSD-95 eGFP	pcDNA3	SV40	CMV	PSD-95	FRET, clustering	eGFP at position +253	2f, 8f, S35f-g	2
p37	SAP97 eGFP	pcDNA3	SV40	CMV	SAP97	FRET	eGFP at position +379	2f	•
p38	PSD-95 C3/5S eGFP	pcDNA3	SV40	CMV	PSD-95 [C3S, C5S]	FRET	eGFP at position +253	2f	•
p39	PSD-93 eGFP	pcDNA3	SV40	CMV	PSD-93	FRET	eGFP at position +282	S5b-c	•
p40	PSD-95 H130V, H372V eGFP	pcDNA3	SV40	CMV	PSD-95 [H130V, H372V]	co-IP	eGFP at position +253	8e	•
p41	pcDNA-TM-mCherry-Xph15	pcDNA3	SV40	CMV	Xph15	FRET	mCherry	2f, S5b-c	•
p42	pcDNA-TM-mCherry-Xph17	pcDNA3	SV40	CMV	Xph17	FRET	mCherry	2f	•
p43	pcDNA-TM-mCherry-Xph18	pcDNA3	SV40	CMV	Xph18	FRET	mCherry	2f, S5b-c	•
p44	pcDNA-TM-mCherry-Xph19	pcDNA3	SV40	CMV	Xph19	FRET	mCherry	2f	•
p45	pcDNA-TM-mCherry-Xph20	pcDNA3	SV40	CMV	Xph20	FRET	mCherry	2f, S5b-c	•
p46	pcDNA-TM-mCherry-Xph24	pcDNA3	SV40	CMV	Xph24	FRET	mCherry	2f	•
p47	pcDNA-TM-mCherry-Xph25	pcDNA3	SV40	CMV	Xph25	FRET	mCherry	2f	•
p48	pcDNA-TM-mCherry-Xph0	pcDNA3	SV40	CMV	Xph0	FRET	mCherry	2f, S5b-c	•
p49	pET-SUMOc-Xph20	pET-SUMOc	pBR322	T7	Xph20	ITC, NMR, SPR, FPLC	TEV_{cs}; SUMO; His_{10}	3c, 5b, S6, S8, S12, S15, S23, S25c	•
p50	pET-SUMOc-Xph18	pET-SUMOc	pBR322	T7	Xph18	ITC, NMR, SPR, FPLC	TEV_{cs}; SUMO; His_{10}	3b, S6, S8, S11, S16, S23, S25c	•
p51	pET-SUMOc-Xph15	pET-SUMOc	pBR322	T7	Xph15	ITC, NMR, SPR, FPLC	TEV_{cs}; SUMO; His_{10}	3a, 5b, S6, S8, S10, S14, S23, S25c	•
p52	pIGb-Xph15	pIGb	pBR322	T7	Xph15 [S63K]	SPR	AviTag; His_{10}	5a, S6	•
p53	pIGb-Xph18	pIGb	pBR322	T7	Xph18 [S63K]	SPR	AviTag; His_{10}	5a, S6	•
p54	pIGb-Xph20	pIGb	pBR322	T7	Xph20 [S63K]	SPR	AviTag; His_{10}	5a, S6, S23b	•
p55	NO-PSD-95-12	pET-NO	pBR322	T7	PSD-95-12 [61-249]	NMR, SPR, Pull Downs, ITC, Photocrosslink	His_8; TEV_{cs}	3, 5, 6b-c, 8b, S8-S12, S26a, S27, S28, S30b, S31-33	•
p56	NO-SAP97-12	pET-NO	pBR322	T7	SAP97-12 [220-408]	NMR, SPR, Pull Downs, Photocrosslink	His_8; TEV_{cs}	5a, 6c, S8, S23b, S26-28, S31	•
p57	NO-PSD-93-12	pET-NO	pBR322	T7	PSD-93-12 [93-282]	NMR, SPR, Pull Downs, Photocrosslink	His_8; TEV_{cs}	5a, 6c, S8, S26-28, S31	•
p58	NO-SAP102-12	pET-NO	pBR322	T7	SAP102-12 [145-333]	NMR, SPR, Pull Downs, Photocrosslink	His_8; TEV_{cs}	5a, 6c, S8, S26-28, S31	•
p59	pET-IG-PSD-95-1	pET-IG	pBR322	T7	PSD-95-1 [61-152]	NMR	His_{10}; TEV_{cs}	S13-S18, S21	•
p60	pET-IG-PSD-95-2	pET-IG	pBR322	T7	PSD-95-2 [155-249]	NMR	His_{10}; TEV_{cs}	S13-S16, S28	•
p61	pbIG-PSD-95-12 [F119R]	pbIG	pBR322	T7	PSD-95-12 [61-249, F119R]	SPR, NMR	AviTag; His-10; TEV_{cs}	S25a,S25c	•
p62	pbIG-SAP97-12 [R278F]	pbIG	pBR322	T7	SAP97-12 [220-408, R278F]	SPR, NMR	AviTag; His-10; TEV_{cs}	S25b	•
p63	pIG-Xph20-ETWV	pET-IG	pBR322	T7	Xph20 [S63K]-$(GGS)_{12}$-ETWV	Pull-Downs, FPLC, MS	His-10; TEV_{cs}	6c, S6, S25b-e, S27b, S29	•
p64	pIG-Xph0-ETWV	pET-IG	pBR322	T7	Xph0-(GGS)12-ETWV	Pull-Downs, FPLC	His-10; TEV_{cs}	6c, S6, S25f-g	•
p65	pbIG-Xph15-ETWV	pbIG	pBR322	T7	Xph15 [S63K]-(GGS)12-ETWV	SPR	AviTag; His_{10}; TEV_{cs}	6b, S26	•
p66	pbIG-Xph18-ETWV	pbIG	pBR322	T7	Xph18 [S63K]-(GGS)12-ETWV	SPR	AviTag; His_{10}; TEV_{cs}	6b, S26	•
p67	pbIG-Xph20-ETWV	pbIG	pBR322	T7	Xph20 [S63K]-(GGS)12-ETWV	SPR, Pull-Down	AviTag; His_{10}; TEV_{cs}	6b, 7a, 7c, S26, S30a	•
p68	pbIG-Xph0-ETWV	pbIG	pBR322	T7	Xph0-(GGS)12-ETWV	Pull-Down	AviTag; His_{10}; TEV_{cs}	7d	•
p69	pbIG-Xph18-Stg	pbIG	pBR322	T7	Xph18 [S63K]-Stg	SPR	AviTag; His_{10}; TEV_{cs}	6b	•
p70	pbIG-Xph20-Stg	pbIG	pBR322	T7	Xph20 [S63K]-Stg	SPR	AviTag; His_{10}; TEV_{cs}	6b	•
p71	pIG-Xph20-ETWV*	pET-IG	pBR322	T7	Xph20 [S63K]-(GGS)12-ETWV [-5 TAG, G-11 R]	Photocrosslink	His_{10}; TEV_{cs}	8b, S31, S32, S33, S34	•
p72	pIG-Xph20-Stg*	pET-IG	pBR322	T7	Xph20 [S63K]-Stg [-5 TAG, L-12 R]	Photocrosslink	His_{10}; TEV_{cs}	8b, S31, S32	•
p73	pEVOL-pAzF	p15A	p15A	araBAD	M.j. p-azidophenylalanine RS (2 copies + tRNA)	Photocrosslink	—	8b, S31, S32, S33	3
p74	pCAG-mIRFP670nuc-TEV-Xph20-ETWV	pCAG	SV40	CAG	Xph20 [S63K]-ETWV, mIRFP670-Nuc	FRET, co-IP, clustering	—	8d-f, S35	•
p75	pCAG-mIRFP670nuc-TEV-Xph0-ETWV	pCAG	SV40	CAG	Xph0-ETWV, mIRFP670-Nuc	FRET, co-IP, clustering	—	8d-f, S35	•
p76	pCAG-mIRFP670nuc-TEV-Xph20	pCAG	SV40	CAG	Xph20 [S63K], mIRFP670-Nuc	FRET, co-IP, clustering	—	8e-f, S35	•
p77	pcDNA-FRET-PSD-95-12(H130V, no stg)	pcDNA3	SV40	CMV	FRET sensor OFF (negative control)	FRET	eGFP, mCherry	8d, S35	•
p78	pcDNA-FRET-PSD-95-12(H130V)	pcDNA3	SV40	CMV	FRET sensor	FRET	eGFP, mCherry	8d, S35	•
p79	pIGc-Xph20	pIGc	pBR322	T7	Xph20 [S63K]	NMR, Unfolding, SPR	His_{10}	S7, S18, S20, S21, S25, S27	•
p80	pIGc-Xph18	pIGc	pBR322	T7	Xph18 [S63K]	NMR, Unfolding, ITC	His_{10}	5b, S7, S21, S25	•
p81	pIGc-Xph15	pIGc	pBR322	T7	Xph15 [S63K]	NMR, Unfolding	His_{10}	S7, S17,S19, S25	•
p82	pIGb-Xph15	pIGb	pBR322	T7	Xph15	FPLC	AviTag; His_{10}; TEV_{cs}	5a, S6	•
p83	pIGb-Xph18	pIGb	pBR322	T7	Xph18	FPLC	AviTag; His_{10}; TEV_{cs}	5a, S6	•
p84	pIGb-Xph20	pIGb	pBR322	T7	Xph20	FPLC	AviTag; His_{10}; TEV_{cs}	5a, S6, S23b	•
p85	HA-Stargazin mCherry	pcDNA3	SV40	CMV	Stargazin	co-IP	HA in loop 1, mCherry at position -21	8e, S35	•
p86	pDisplay-BirA-ER	pDisplay	SV40	CMV	BirA-ER	Clustering	—	8f, S35	4
p87	AP-LRRTM2	pBOS	SV40	human EF-1α	LRRTM2	Clustering	Avitag	8f, S35	5
p88	pIG-Xph20-ETMA*	pET-IG	pBR322	T7	Xph20[S63K]-(GGS)12-ETMA [-5 TAG, G-11 R]	Photocrosslink	His_{10}; TEV_{cs}	S33, S34	•
p89	pIG-TAT-Xph20-ETWV	pET-IG	pBR322	T7	TAT-Xph20 [S63K]-(GGS)12-ETWV	co-IP	His_{10}; TEV_{cs}	7b, S30c-d	•

Supplementary Table 10 | Primers used in this study.

Name	Seq (5'-3')	Purpose	Used for plasmid
Xop-0502	cattgcggccgcgggcagctctgtcag	Xph0 cloning in pSEX	p02
Xop-0503	ccgaccggtacggtaattgatag	Xph0 cloning in pSEX	p02
Xop-0890	cggtcatgaatgtgagcaagggcgaggagg	mCh-BirA cloning in pACYC-duet-1	p14
Xop-0873	ccgctcgaggagctcttatttttctgcactacgc	mCh-BirA cloning in pACYC-duet-1	p14
Xop-0124	cgggatccatggagtatgaggagatcacattgg	PSD-95-12 and PSD-95-1 cloning in pbIG, pET-IG, pET-NO	p16, p59, p55
Xop-0233	ccgctcgagttaggcattgctgggcttggccaccttt	PSD-95-12 and PSD-95-1 cloning in pbIG, pET-IG, pET-NO	p16, p60, p55
Xop-0892	cgggatcctatgaatatgaggaaatcacac	SAP97-12 cloning in pbIG, pET-NO	p17, p56
Xop-0895	ccgctcgagttacatacttgttggttttgccac	SAP97-12 cloning in pbIG, pET-NO	p17, p56
Xop-0956	ctggatccttcaagtatgaggagatagtcctg	SAP102-12 cloning in pbIG, pET-NO	p18, p58
Xop-0957	gtctcgagttactgtcgcctccgcaccaccaaccg	SAP102-12 cloning in pbIG, pET-NO	p18, p58
Xop-0080	cgggatccgaatatgaatttgaagaaattacattgg	PSD-93-12 cloning in pbIG, pET-NO	p19, p57
Xop-0055	ccgctcgagttaaatggttgtgggtttgccaac	PSD-93-12 cloning in pbIG, pET-NO	p19, p57
Xop-0230	cgggatccgccgaaaaggtcatggagatc	PSD-95-2 cloning in pbIG, pET-IG	p20, p60
Xop-0233	ccgctcgagttaggcattgctgggcttggccaccttt	PSD-95-2 cloning in pbIG, pET-IG	p20, p60
Xop-0266	cgggatccggcagctctgtcagttcc	Xph cloning in pET-IG, pbIG	p21-29, p63-72
Xop-0267	ccctcgagttaggtacggtaattgatagaaatc	Xph cloning in pET-IG	p21-29
Xop-0050	cgggatccgagaatttgtattttcagggctatgaatttgaagaaattac	PSD-93-12 cloning in pGEX-4T-2	p33
Xop-0049	ccgctcgagttacttatcgtcatcgtctttgtagtcaatggttgtgggtttgccaac	PSD-93-12 cloning in pGEX-4T-2	p33
GFP 15F	ctcgagatggtgagcaagggcgag	EGFP cloning in pcDNA-SAP97	p37
YFP 6R	gctagccttgtacagctcgtccatgcc	EGFP cloning in pcDNA-SAP97	p37
PSD95_C3/5S_F	gcttcccaacatggactctctctatagtgacaaccaagaaatacc	site directed mutagenesis (PSD-95 C3S/C5S)	p38
PSD95_C3/5S_R	ggtatttcttggttgtcactatagagagagagtccatgttgggaagc	site directed mutagenesis (PSD-95 C3S/C5S)	p38
Xop-1022	cggaagcttgccaccatgatttgccactgcaaagttg	PSD-93 cloning in pcDNA3	p39
Xop-1007	cggctcgagattataacttctcctttgaggg	PSD-93 cloning in pcDNA3	p39
Xop-1008	cggtgtacaagactagttatatgactgatccttatgg	PSD-93 cloning in pcDNA3	p39
Xop-1005	cggaccggtaatggttgtgggtttgccaac	PSD-93 cloning in pcDNA3	p39
Xom-0026	gtggatgttcgggaggtgaccgtttccgctgcggtggaggccctc	site directed mutagenesis (PSD-95 H130V)	p40
Xom-0027	gagggcctccaccgcagcggaaacggtcacctcccgaacatccac	site directed mutagenesis (PSD-95 H130V)	p40
Xom-0050	gttgacctccgcaatgccagtgtcgaacaggctg	site directed mutagenesis (PSD-95 H372V)	p40
Xom-0051	cagcctgttcgacactggcattgcggaggtcaac	site directed mutagenesis (PSD-95 H372V)	p40
Xop-0612	gggaattccatatgagctctgtcagttccgtgccg	Xph cloning in pET-SUMOc, pIGb, pIGc	p49-54, p79-84
Xop-0583	ccgctcgagggtaccggtacggtaattg	Xph cloning in pET-SUMOc, pIGb, pIGc	p49-54, p68, p79-84
Xom-0166	tatagtcgacacccggtttcaggccggaaatcgttg	site directed mutagenesis (Xph S63K)	p52-54, p79-81
Xom-0167	caacgatttccggcctgaaaccgggtgtcgactata	site directed mutagenesis (Xph S63K)	p52-54, p79-81
Xop-0231	ccgctcgagttatttccggcgcatgacatag	PSD-95-1 cloning in pbIG, pET-IG	p59
Xop-0230	cgggatccgccgaaaaggtcatggagatc	PSD-95-2 cloning in pbIG, PET-IG	p60
Xom-0174	gggtcaatgacagcatcctgcgtgtaaatgaagtggatgttc	site directed mutagenesis (PSD-95 F119R)	p61
Xom-0179	gaacatccacttcatttacacgcaggatgctgtcattgaccc	site directed mutagenesis (PSD-95 F119R)	p61
Xom-0176	catcacgaacatctgcttcatttacgaacagtatacagtcatttacccgcaat	site directed mutagenesis (SAP97 R278F)	p62
Xom-0177	attgcgggtaaatgactgtatactgttcgtaaatgaagcagatgttcgtgatg	site directed mutagenesis (SAP97 R278F)	p62
Xop-0493	ccctcgagggtacggtaattgatagaaatc	Xph cloning in pET-IG, pbIG	p63, p65-67, p71
Xop-0583	ccgctcgagggtaccggtacggtaattg	Xph0 cloning in pET-IG, pbIG	p64, p68
Xop-0795	ccctcgagtcccgaaccgcccgaaccggtacggtaattgatagaaatc	Xph cloning in pET-IG, pbIG	p69-70, p72
Xom-0202	gccggaaccacggctgcctccgc	site directed mutagenesis (Xph-ETWV -11 Arg)	p71
Xom-0201	gcggaggcagccgtggttccggc	site directed mutagenesis (Xph-ETWV -11 Arg)	p71
Xom-0198	gtggttccggcggaagctagaccgaaacctgggtgta	site directed mutagenesis (Xph-ETWV -5 TAG)	p71
Xom-0197	tacacccaggtttcggtctagcttccgccggaaccac	site directed mutagenesis (Xph-ETWV -5 TAG)	p71
Xom-0204	ggcagtattcgcatgccgggatgaaccactaga	site directed mutagenesis (Xph-Stg -12 Arg)	p72
Xom-0203	tctagtggttcatcccggcatgcgaatactgcc	site directed mutagenesis (Xph-Stg -12 Arg)	p72
Xom-0200	ccctgcatgcgaatactgccaattagcgcacgacaccg	site directed mutagenesis (Xph-Stg -5 TAG)	p72
Xom-0199	cggtgtcgtgcgctaattggcagtattcgcatgcaggg	site directed mutagenesis (Xph-Stg -5 TAG)	p72
Xop-0990	ggcgctagctccgtgccgaccaaactg	Xph cloning in pCAG	p76
Xop-1057	ccgtgtacattaacggtaattgatagaaatc	Xph cloning in pCAG	p76
Xom-0222	agctagaccgaaaccatggcgtaagtcgacgcggc	site directed mutagenesis (Xph-ETWV to -ETMA)	p88
Xom-0223	gccgcgtcgacttacgccatggtttcggtctagct	site directed mutagenesis (Xph-ETWV to -ETMA)	p88
Xos-0024	cactttatgcttccggctcg	pSEX sequencing	p02-13

Supplementary Table 11 | Full amino acid sequence details of main constructs. In Xph constructs, the 63 position is underlined. The template used for selection yielded sequences with a serine at that position, which was later mutated to lysine to improve solubility.

PROTEIN NAME (GENE)	AMINO ACID SEQUENCE
**PSD-95-12 (DLG4)**	MEYEEITLERGNSGLGFSIAGGTDNPHIGDDPSIFITKIIPGGAAAQDGRLRVNDSILFVNEVDVREVTHSAAVEALKEAGSIVRLYVMRR<br>KPPAEKVMEIKLIKGPKGLGFSIAGGVGNQHIPGDNSIYVTKIIEGGAAHKDGRLQIGDKILAVNSVGLEDVMHEDAVAALKNTYDVVYLKVAKPSNA
**SAP97-12 (DLG1)**	YEYEEITLERGNSGLGFSIAGGTDNPHIGDDSSIFITKIITGGAAAQDGRLRVNDCILRVNEADVRDVTHSKAVEALKEAGSIVRLYVKRR<br>KPASEKIMEIKLIKGPKGLGFSIAGGVGNQHIPGDNSIYVTKIIEGGAAHKDGKLQIGDKLLAVNSVCLEEVTHEEAVTALKNTSDFVYLKVAKPTSM
**PSD-93-12 (DLG2)**	EYEFEEITLERGNSGLGFSIAGGTDNPHIGDDPGIFITKIIPGGAAAEDGRLRVNDCILRVNEVDVSEVSHSKAVEALKEAGSIVRLYVRRR<br>RPILETVVEIKLFKGPKGLGFSIAGGVGNQHIPGDNSIYVTKIIDGGAAQKDGRLQVGDRLLMVNNYSLEEVTHEEAVAILKNTSDVVYLKVGKPTTI
**SAP102-12 (DLG3)**	FKYEEIVLERGNSGLGFSIAGGIDNPHVPDDPGIFITKIIPGGAAAMDGRLGVNDCVLRVNEVDVSEVVHSRAVEALKEAGPVVRLVVRRR<br>QPPPETIMEVNLLKGPKGLGFSIAGGIGNQHIPGDNSIYITKIIEGGAAQKDGRLQIGDRLLAVNNTNLQDVRHEEAVASLKNTSDMVYLKVAKPGSL
**Xph15**	SSVSSVPTKLEVVAATPTSLLISWDAGPRNVSYYRITYGETGGNSPVQEFTVPGSSSTATISGL<u>S</u>PGVDYTITVYASGHVSTLMTPISINYRT
**Xph18**	SSVSSVPTKLEVVAATPTSLLISWDAHSRMAWVNYYRITYGETGGNSPVQEFTVPGSSSTATISGL<u>S</u>PGVDYTITVYANGGGFRRGMAPISINYRT
**Xph20**	SSVSSVPTKLEVVAATPTSLLISWDAVAKNVKVGYYRITYGETGGNSPVQEFTVPGSSSTATISGL<u>S</u>PGVDYTITVYANGVLSKMVLPISINYRT
**Xph20-ETWV**	SSVSSVPTKLEVVAATPTSLLISWDAVAKNVKVGYYRITYGETGGNSPVQEFTVPGSSSTATISGL<u>K</u>PGVDYTITVYANGVLSKMVLPISINYRTLE<br>GGSGGSGGSGGSGGSGGSGGSGGSGGSGGSGGSGGSGTETWV
**Xph20-Stg**	SSVSSVPTKLEVVAATPTSLLISWDAVAKNVKVGYYRITYGETGGNSPVQEFTVPGSSSTATISGL<u>K</u>PGVDYTITVYANGVLSKMVLPISINYRT<br>GSGGSGLEGSSGSSGSSGSSGSSGSSLHANTANRRTTPV

Supplementary Note 1 | Specificity vs selectivity

Specificity: we use the term to refer to the fact that a given selected clone recognizes an epitope that is unique to the targeted protein (PSD-95) resulting in the absence of detectable binding to the other family members (SAP97, SAP102 and PSD-93) using a set of experimental approaches and concentration range.

Selectivity: we use the term to refer to the engineered competitors that can all bind to class I PDZ domain-containing proteins -and in particular to the four DLG tandem PDZ domains- via the binding motif (-ETWV or -Stg) but due to the fusion to Xph15, Xph18 or Xph20 bind much strongly and preferentially to PSD-95 tandem PDZ domains.

Supplementary Note 2 | Degenerated oligonucleotides used for the library generation.

BC loop oligonucleotides:

5′-P-cggggagttaccgcccgtttcgccataggtgatacggtaatamnngac[mnn]$_{4-8}$tgcatcccagctgatcagcagagacgtcggggtgcgcgc

FG loop oligonucleotides:

5′-P-gcccgaaccgccggtaccggtacggtaattgataatcgg[mnn]$_{7-12}$agcgtaaaccgtaatggtatagtcgacacccggtgacaggcc

Supplementary Note 3 | KingFisherDuo protocol for the competitive pull-down assay.

The titrations were performed using a KingFisher Duo system in 96 deep-well plates.

The plate were prepared as follow:

(A) Tip Comb.
(B) 50 µL of Dynabeads. Add PT to the final volume of 100 µL (PT: PBS at 500mM NaCl, pH 7.4 + 0.01% Tween-20).
(C) 1000 µL of PT-BSA (0.5% BSA).
(D) 120 pmoles of biotinylated Stargazin divalent ligand. Add PT to the final volume of 100 µL.
(E) 1000 µL of PT-BSA.
(F) 180 pmoles of purified tandem PDZ domains
(G) Different molar ratio of competitor (Xph20 or Xph0 fused to the ETWV motif) in a final PT-BSA volume of 1000 µL.
(H) 1000 µL of PT.

The KingFisher protocol was set up as follow:

1. Load tip comb from (A)
2. Mix the beads for 1 min 30 then collect for 30 s 5 times (B).
3. Release the beads into (C), mix at fast speed for 1 min 30 and collect for 10 s 5 times.
4. Release the beads into (D), mix at fast speed for 1 min 30 and collect for 10 s 5 times.
5. Release the beads into (E), mix at fast speed for 1 min 30 and collect for 10 s 5 times.
6. Release the beads into (F), mix at medium speed for 6 min and collect for 30 s 5 times.
7. Release the beads into (G), mix at medium speed for 6 min and collect for 30 s 5 times.
8. Release the beads into (H), mix at fast speed for 30 sec and drop the tip comb back into (A).

The mix from the masterblock (H) wells were transferred into new 1.5 mL LoBind tubes and the beads recovered for SDS-PAGE analysis.

Supplementary References

1. Sainlos M, Iskenderian WS, Imperiali B. A general screening strategy for peptide-based fluorogenic ligands: probes for dynamic studies of PDZ domain-mediated interactions. *J. Am. Chem. Soc.* **131**, 6680-6682 (2009).

2. Sainlos M*, et al.* Biomimetic divalent ligands for the acute disruption of synaptic AMPAR stabilization. *Nat. Chem. Biol.* **7**, 81-91 (2011).

3. Chin JW, Santoro SW, Martin AB, King DS, Wang L, Schultz PG. Addition of p-azido-L-phenylalanine to the genetic code of Escherichia coli. *J. Am. Chem. Soc.* **124**, 9026-9027 (2002).

4. Howarth M*, et al.* Monovalent, reduced-size quantum dots for imaging receptors on living cells. *Nat. Methods* **5**, 397-399 (2008).

5. Chamma I*, et al.* Mapping the dynamics and nanoscale organization of synaptic adhesion proteins using monomeric streptavidin. *Nat. Commun.* **7**, 10773 (2016).
