# Supplementary material for: Engineering selective competitors for the discrimination of highly conserved protein-protein interaction modules
Source: Nat Commun. 2019 Oct 4;10:4521. doi: 10.1038/s41467-019-12528-4 (PMC6778148; doi:10.1038/s41467-019-12528-4)
Supplement: Supplementary file 3 — Reporting Summary [file 41467_2019_12528_MOESM3_ESM.pdf]

nature research

Corresponding author(s): Matthieu Sainlos
Cameron Mackereth

Last updated by author(s): Sep 9, 2019

Reporting Summary

Nature Research wishes to improve the reproducibility of the work that we publish. This form provides structure for consistency and transparency in reporting. For further information on Nature Research policies, see Authors & Referees and the Editorial Policy Checklist.

Statistics

For all statistical analyses, confirm that the following items are present in the figure legend, table legend, main text, or Methods section.

n/a	Confirmed	
☐	☒	The exact sample size (*n*) for each experimental group/condition, given as a discrete number and unit of measurement
☐	☒	A statement on whether measurements were taken from distinct samples or whether the same sample was measured repeatedly
☐	☒	The statistical test(s) used AND whether they are one- or two-sided<br>*Only common tests should be described solely by name; describe more complex techniques in the Methods section.*
☒	☐	A description of all covariates tested
☒	☐	A description of any assumptions or corrections, such as tests of normality and adjustment for multiple comparisons
☐	☒	A full description of the statistical parameters including central tendency (e.g. means) or other basic estimates (e.g. regression coefficient) AND variation (e.g. standard deviation) or associated estimates of uncertainty (e.g. confidence intervals)
☒	☐	For null hypothesis testing, the test statistic (e.g. *F*, *t*, *r*) with confidence intervals, effect sizes, degrees of freedom and *P* value noted<br>*Give P values as exact values whenever suitable.*
☒	☐	For Bayesian analysis, information on the choice of priors and Markov chain Monte Carlo settings
☒	☐	For hierarchical and complex designs, identification of the appropriate level for tests and full reporting of outcomes
☒	☐	Estimates of effect sizes (e.g. Cohen's *d*, Pearson's *r*), indicating how they were calculated

Our web collection on statistics for biologists contains articles on many of the points above.

Software and code

Policy information about availability of computer code

Data collection	TopSpin v4.0, Metamorph v7.8.10.0, LI-FLIM v1.2.12, LI-COR Image Studio v5.2, POLARstar Omega v5.11, Bio-Rad Image Lab v5.0
Data analysis	SynDivA (https://services.cbib.u-bordeaux.fr/galaxy/tool_runner?tool_id=fibronectin), Microsoft Office 2016, GraphPad Prism v7, Clustal Omega, CARA v1.9.1.3, NMRPipe v8.6, Sparky v3.113, MODULE2, PyMol v2, Bio-Rad Image Lab v5.0, Biacore Evaluation software v3.1, StavroX v3.6.6, Fiji v2, LI-FIM v1.2.12, LI-COR Image Studio v5.2, POLARstar MARS data analysis software v3.20

For manuscripts utilizing custom algorithms or software that are central to the research but not yet described in published literature, software must be made available to editors/reviewers. We strongly encourage code deposition in a community repository (e.g. GitHub). See the Nature Research guidelines for submitting code & software for further information.

Data

Policy information about availability of data

All manuscripts must include a data availability statement. This statement should provide the following information, where applicable:

- Accession codes, unique identifiers, or web links for publicly available datasets
- A list of figures that have associated raw data
- A description of any restrictions on data availability

Backbone 1H, 13C and 15N chemical shift assignments for PSD-95-12, PSD-95-1 and PSD-95-2 were deposited in the Biological Magnetic Resonance Data Bank (BMRB) as entries 27308, 27309 and 27310, respectively. The mass spectrometry proteomics data have been deposited to the ProteomeXchange Consortium via the PRIDE partner repository with the dataset identifiers PXD015313 (cellular target identification) and PXD015366 (photocrosslinking). The authors declare that the data supporting the findings of this study are available from the corresponding authors upon reasonable request.

Field-specific reporting

Please select the one below that is the best fit for your research. If you are not sure, read the appropriate sections before making your selection.

☒ Life sciences ☐ Behavioural & social sciences ☐ Ecological, evolutionary & environmental sciences

For a reference copy of the document with all sections, see nature.com/documents/nr-reporting-summary-flat.pdf

Life sciences study design

All studies must disclose on these points even when the disclosure is negative.

Sample size	No method was used to determine sample sizes and sample sizes were based upon numbers used inn similar published studies
Data exclusions	No data was excluded for analysis
Replication	All attempts of replication were successful. Experiments were reliably repeated in independent replicates, as indicated in the figures, the figure legends or the methods
Randomization	No data randomization was performed
Blinding	No blinding was applied

Reporting for specific materials, systems and methods

We require information from authors about some types of materials, experimental systems and methods used in many studies. Here, indicate whether each material, system or method listed is relevant to your study. If you are not sure if a list item applies to your research, read the appropriate section before selecting a response.

Materials & experimental systems

n/a	Involved in the study	
☐	☒	Antibodies
☐	☒	Eukaryotic cell lines
☒	☐	Palaeontology
☐	☒	Animals and other organisms
☒	☐	Human research participants
☒	☐	Clinical data

Methods

n/a	Involved in the study	
☒	☐	ChIP-seq
☒	☐	Flow cytometry
☒	☐	MRI-based neuroimaging

Antibodies

Antibodies used	Phage-ELISA: anti-M13 monoclonal antibody (GE Healthcare, cat # 27-9420-01, 1/5,000)<br>Co-IP and Western Blots: anti-His antibody (Abcam, cat # ab18184), anti-PSD-95 antibody (Cell Signalling, cat # 2507), anti-PSD-95 (Millipore-Merck, cat # MAB1596, 1/1,000), anti-Histidine tag (Sigma-Aldrich, cat # H1029, 1/1,000), anti-mouse IRDye-680LT (LI-COR, cat # 926-68020, 1/15,000) and anti-mouse IgG, Fcγ fragment specific Alexa Fluor 790 (Jackson ImmunoResearch, cat # 115-655-071, 1/15,000), anti-mouse light chain IRDye-800 (Jackson ImmunoResearch, cat # 115-655-174, 1/15,000), anti-GFP (Abcam, cat # ab290, 1/5,000), anti-mCherry (Abcam, cat # ab167453, 1/1,000), anti-rabbit IgG, light chain specific IRDye-800W secondary antibody (Jackson Immunoresearch, cat # 211-652-171, 1/15,000)
Validation	All antibodies used were commercial and validated by the manufacturer.

Eukaryotic cell lines

Policy information about cell lines

Cell line source(s)	Human embryonic kidney (HEK)293T cells (ATCC CRL-3216), COS-7 cells (ECACC-87021302)
Authentication	Each cell line came directly from commercial sources that state that these cell lines are authentic. We did not do any inhouse identification post arrival of aliquots.
Mycoplasma contamination	All cell lines were tested negative for mycoplasma contamination.
Commonly misidentified lines<br>(See ICLAC register)	None of the cell lines used in this study are in the database of commonly misidentified cell lines.

Animals and other organisms

Policy information about studies involving animals; ARRIVE guidelines recommended for reporting animal research

Laboratory animals	Adult (2-3 months old) Sprague-Dawley rats raised in the animal facility of Bordeaux University B 33 063 917
Wild animals	*Provide details on animals observed in or captured in the field; report species, sex and age where possible. Describe how animals were caught and transported and what happened to captive animals after the study (if killed, explain why and describe method; if released, say where and when) OR state that the study did not involve wild animals.*
Field-collected samples	*For laboratory work with field-collected samples, describe all relevant parameters such as housing, maintenance, temperature, photoperiod and end-of-experiment protocol OR state that the study did not involve samples collected from the field.*
Ethics oversight	Experiments were performed in accordance with the European 2010/63/EU directive and approved by the Bordeaux University Ethics Committee (CE50)

Note that full information on the approval of the study protocol must also be provided in the manuscript.
